# Challenges and drivers in the implementation of current practices in outpatient parenteral antimicrobial therapy: an international survey

**DOI:** 10.1093/jacamr/dlag095

**Published:** 2026-05-26

**Authors:** Solomon Ahmed Mohammed, Jason A Roberts, David Nicolás, Abdullah Tarik Aslan, Abiola Senok, Laila Aldabal, Daniel Erku, Afra Nahdia Marizan Nor, James Pollard, Menino Cotta, R Andrew Seaton, Mark Gilchrist, Fekade B Sime

**Affiliations:** UQ Centre for Clinical Research, The University of Queensland, Building 71/918 RBWH Herston, Brisbane, Queensland 4006, Australia; Department of Pharmacy, Wollo University, Dessie, Ethiopia; UQ Centre for Clinical Research, The University of Queensland, Building 71/918 RBWH Herston, Brisbane, Queensland 4006, Australia; Department of Pharmacy and Intensive Care Medicine, Royal Brisbane and Women’s Hospital, Brisbane, Queensland, Australia; Herston Infectious Disease Institute (HeIDI), Metro North Health, Queensland, Australia; Division of Anaesthesiology Critical Care Emerging and Pain Medicine, Nimes University Hospital, University of Montpellier, Nîmes, France; Hospital at Home Unit, Medical and Nurse Direction, Hospital Clínic de Barcelona, Universitat de Barcelona, Barcelona, Spain; UQ Centre for Clinical Research, The University of Queensland, Building 71/918 RBWH Herston, Brisbane, Queensland 4006, Australia; College of Medicine, Mohammed Bin Rashid University of Medicine and Health Sciences, Dubai, United Arab Emirates; School of Dentistry, Cardiff University, Cardiff, UK; Rashid Hospital, Dubai Health, Dubai, United Arab Emirates; Centre for Applied Health Economics, Griffith University, Brisbane, Queensland, Australia; Economic, Policy and Innovation Centre for Health Systems (EPIC Health Systems), Addis Ababa, Ethiopia; Pharmacy Department, Hospital Sungai Buloh, Selangor, Malaysia; Cabrini @ Home, Cabrini Health, Melbourne, Australia; UQ Centre for Clinical Research, The University of Queensland, Building 71/918 RBWH Herston, Brisbane, Queensland 4006, Australia; Department of Infectious Diseases, Queen Elizabeth University Hospital, Glasgow, UK; OPAT Initiative, British Society for Antimicrobial Chemotherapy (BSAC), Birmingham, UK; OPAT Initiative, British Society for Antimicrobial Chemotherapy (BSAC), Birmingham, UK; Department of Pharmacy/Infection, Imperial College Healthcare NHS Trust, London, UK; Department of Infectious Diseases, Imperial College, London, UK; UQ Centre for Clinical Research, The University of Queensland, Building 71/918 RBWH Herston, Brisbane, Queensland 4006, Australia; School of Pharmacy and Pharmaceutical Sciences, The University of Queesnland, Brisbane, Australia

## Abstract

**Background:**

Outpatient parenteral antimicrobial therapy (OPAT) is practised worldwide due to the benefits it provides to patients and healthcare systems. However, its full potential remains unrealized due to different implementation challenges. This study aims to identify barriers and facilitators that influence the routine implementation of current practices in the OPAT programme.

**Methods:**

An international, multi-centre electronic survey was conducted among healthcare facilities providing OPAT services in Australia, Malaysia, the UK, Spain, Turkey and Middle East countries. The survey instrument was developed based on the Consolidated Framework for Implementation Research and guideline recommendations for OPAT. Statistical analyses were performed using SPSS version 30.

**Results:**

The survey received responses from 150 healthcare facilities across 10 countries offering OPAT services. The majority (11 879.7%) of healthcare facilities implement OPAT through a formal structure. The majority (92.2%) had a designated team lead. Most facilities (12 685.7%) reported the implementation of antimicrobial toxicity monitoring. Only 58 facilities (39.5%) reported implementation of regular audits of their OPAT programmes. The most reported barriers to OPAT implementation included wide geographic distribution of patients (50.7%), lack of financial support (42.7%) and the dosing frequency of antimicrobials (40.6%). Facilitators to OPAT implementation include hospital bed savings, clinical safety and efficacy, cost-effectiveness and patient satisfaction.

**Conclusions:**

The majority of healthcare facilities implement OPAT through a formal structure. However, several challenges continue to hinder its routine implementation. Ongoing efforts to address implementation barriers are crucial for strengthening existing services and supporting the expansion of new services.

## Introduction

Outpatient parenteral antimicrobial therapy (OPAT) was first introduced in the USA in 1974.^1^ Since its inception, OPAT has evolved substantially into a global practice, becoming an integral component of routine patient care, particularly in developed countries.^2,3^ It is implemented through either diverse clinical settings, including infusion centres, hospital-based ambulatory-care clinics and skilled nursing facilities or patients’ homes.^4^

The scope of OPAT service has been expanding to include a wide range of infections and is now increasingly recognized in contemporary expert recommendations and treatment guidelines.^5^ OPAT has demonstrated safety and favourable clinical outcomes in treating a variety of infections.^6^ The key is patient selection and a multispecialty clinical infection management plan.^7^ Infections such as musculoskeletal, skin and soft-tissue, bone and joint, urinary tract, surgical site, endovascular and bacteraemia are commonplace in programmes.^8^

OPAT programmes offer multifaceted benefits.^9^ Treatment through OPAT has been shown to accelerate both psychological and physical recovery^10,11^ and is associated with high patient satisfaction and preference.^4^ Furthermore, OPAT programmes address the flow and capacity challenges by avoiding unnecessary inpatient days and result in substantial cost savings, reducing patient care expenses by an average of 36%.^12^

Increasing recognition of the advantages that OPAT programmes offer to both the healthcare system and patients has led to expanding uptake and implementation in many countries. However, there is clear heterogeneity in the management and delivery of OPAT services.^13^ Variations in practices can be attributed to a range of factors, including organizational and financial constraints, variations in local health delivery systems, geographical locations and the availability of specialist services.^13–16^

To date, most studies evaluating OPAT practices have focused on individual countries, such as the UK,^17^ Ireland,^18^ France,^16^ the Netherlands^19^ and the USA.^20–22^ International evaluations of OPAT implementation remain limited. A 2004 intercountry comparison involving the USA, Italy and the UK primarily examined disease management and clinical outcomes.^13^ In 2017, another survey explored the nature and extent of OPAT services across 17 Asian countries,^23^ while a 2022 survey evaluated the structure and implementation of OPAT programmes in 34 European countries, though it focused only on the national level without service-specific details.^15^ In 2023, another global survey evaluated the practice and challenges of OPAT implementation; however, the study focused on the perspective of health professionals, mainly pharmacists, and did not comprehensively identify challenges and enablers.^24^ More recently, a 2025 global survey examined the stability of antimicrobials and infusion practices in OPAT service centres.^8^ However, barriers and facilitators to implementing the OPAT programme have not yet been thoroughly studied. There is still a limited understanding of how current healthcare dynamics affect OPAT implementation. This study, therefore, aims to assess OPAT implementation and identify the key barriers and facilitators influencing its implementation.

## Materials and methods

An international, multi-centre electronic survey was conducted from October 2024 to May 2025 across healthcare facilities providing OPAT services in Australia, the UK, Spain, Malaysia, Turkey and Middle Eastern countries, including Saudi Arabia, Oman, Qatar, the United Arab Emirates and Kuwait. Countries were chosen based on pre-existing networks with national coordinators or professional associations that could support survey distribution aiming to cover countries with both emerging and well-established OPAT services. Health service facilities providing OPAT services within participating countries were eligible for inclusion. These facilities were identified and contacted directly through professional associations or national OPAT coordinators.

The survey tool was developed through a multi-step process. First, we reviewed existing literature to identify barriers and facilitators to OPAT implementation, organizing them according to the domains of the Consolidated Framework for Implementation Research (CFIR).^25^ The CFIR is used to evaluate factors influencing the implementation of programmes such as OPAT across five domains: innovation, outer setting, inner setting, characteristics of individuals and process.^26^ Second, we identified recommendations for the optimal OPAT service using the UK OPAT good practice recommendation and categorized them into CFIR domains.^5^ Third, we compared these findings with previously published surveys.^16–22,27,28^ Fourth, we formulated questions from the identified and compared topics. Fifth, we included general questions about the characteristics of OPAT programmes. The survey tool was then reviewed and revised by an infectious disease (ID) physician and collaborators not involved in its development to ensure content validity. The final tool (Supplementary information A, available as Supplementary data at *JAC-AMR* Online) consists of three sections: (i) the demographic part of the survey, (ii) characteristics of the OPAT programme and (iii) barriers and facilitators to implementing the OPAT programme. Barriers and facilitators were rated on a scale from 0 (completely disagree) to 10 (completely agree).

The survey was distributed electronically using the Research Electronic Data Capture (REDCap) platform (Vanderbilt University, Nashville, TN, USA). Participants were invited via email through professional networks, including the British Society for Antimicrobial Chemotherapy, the Hospital-in-the-Home Society Australasia and the Spanish Society of Home Hospitalization. In Turkey, Malaysia and the Middle Eastern countries, OPAT leaders were contacted directly through national coordinators. OPAT leaders were encouraged to collaborate with their teams to complete the survey collectively, submitting a single response per OPAT centre. Follow-up emails were sent to non-respondents at 4, 8 and 12 weeks after the initial invitation, each scheduled on different days.

The collected data were directly imported into SPSS version 30 (IBM, Chicago, IL, USA) for analysis. Responses were included in the analyses if the first section of the questionnaire was fully completed and at least one question from either Section 2 or Section 3 was answered. Descriptive results are presented as percentages. The denominator used to calculate percentages for the characteristics of health facilities and services was the number of responses received for each characteristic. For the presence of a structured OPAT programme, percentages were calculated using the total number of responses for the respective variable as the denominator. To assess associations between service characteristics and the presence of a formal OPAT structure, χ^2^ tests were performed. Fisher’s exact test was applied when the assumptions for the χ^2^ were not met. A *P* value of <0.05 was considered statistically significant. A formal OPAT service structure is the presence of care coordination with clear clinical and managerial accountabilities of health professionals involved in the OPAT care pathway.^5^

Ethical approval for the study was obtained from the University of Queensland Human Research Ethics Committee (2024/HE000319). Written informed consent was obtained from all participants. Participation was entirely voluntary, and no incentives were provided.

## Results

### Characteristics of health facilities implementing OPAT services

Survey responses were received from 150 health facilities across ten countries that provide OPAT services. The characteristics of these facilities are presented in Table 1. Most responses were from Spain (52, 34.7%), the UK (44, 29.3%) and Australia (18, 12%). The majority of the facilities (72.3%) are located in urban areas, and nearly two-thirds (64.7%) are university teaching hospitals.

**Table 1.** Characteristics of health facilities and their association with the presence of a structured OPAT programme

Characteristics of health facilities			Presence of a structured OPAT programme		
	Frequency	Percent	Frequency	Percent	*P* value
Country (*n* = 150)					
Spain	52	34.7	40	76.9	0.015
UK	44	29.3	35	81.4	
Australia	18	12.0	17	94.4	
Malaysia	16	10.7	15	93.8	
Turkey	13	8.7	5	41.7	
Other^a^	7	1.3	6	85.7	
Location of OPAT centre (*n* = 148)					
Urban	107	72.3	89	84.8	0.133
Suburban	24	16.2	17	70.8	
Rural	17	11.5	12	70.6	
Type of healthcare facility (*n* = 150)					
University teaching hospital	97	64.7	80	83.3	0.092
Community teaching hospital	23	15.3	13	59.1	
Non-teaching hospital	21	14.0	17	81	
Other^b^	9	6.0	8	88.9	

OPAT, outpatient parenteral antimicrobial therapy.

^a^United Arab Emirates, Qatar, Oman, Saudi Arabia and Kuwait.

^b^Community team, general hospital, teaching district general hospital, community admission avoidance rapid response, cure hospital, private independent provider, tertiary military hospital and teaching centre.

The service characteristics of facilities providing OPAT are presented in Table 2. The majority of the facilities (118, 78.7%) provide OPAT services exclusively for adults, while 26 facilities (17.3%) offer both adult and paediatric services. Out of the 150 facilities, 38 (25.3%) manage more than 50 OPAT patients per month. Less than half (44.9%) of the facilities provide a self-administered OPAT (S-OPAT) care model. The majority of health facilities (86.7%) monitor patients for antimicrobial toxicity. The frequency of monitoring antimicrobial toxicity depends on the type of antimicrobials; in half of the facilities, and more than two-thirds of the health facilities (39.7%) monitor patients weekly. The majority of facilities (118, 79.7%) implement OPAT through a formal service structure. The implementation of a formal OPAT structure is significantly associated with the presence of dedicated OPAT team members, integration within an antimicrobial stewardship (AMS) team, training for self-administration, regular audits, structured patient monitoring and follow-up and toxicity monitoring.

**Table 2.** Service characteristics and their association with the presence of a structured OPAT programme

	Service characteristics		Presence of a structured OPAT programme		
	Frequency	Percent	Frequency	Percent	*P* value
OPAT population (*n* = 150)					
Adult OPAT	118	78.7	94	81	0.231
Paediatric OPAT	6	4.0	6	100	
Adult and paediatric OPAT	26	17.3	18	69.2	
Number of patients managed by the OPAT programme monthly (*n* = 150)					
1–5 patients per month	31	20.7	21	70	0.048
6–15 patients per month	21	14.0	17	81	
16–25 patients per month	28	18.7	20	71.4	
26–50 patients per month	32	21.3	24	77.4	
>50 patients per month	38	25.3	36	94.7	
Members of a formal OPAT service structure team (*n* = 118)					
Infectious diseases physician	81	68.6	NA		
Clinical pharmacist	79	66.9			
Specialist nurse	69	58.5			
Acute physician	58	49.2			
Administrative assistant	44	37.3			
Clinical microbiologist	38	32.2			
Community nurse	35	29.7			
Advanced nurse practitioner	30	25.4			
Consultant paediatrician	10	8.5			
IT specialist	5	4.2			
Other staff^a^	27	22.9			
Clinical leader of a formal OPAT service structure team (*n* = 118)					
No formal lead	8	6.8	NA		
Infectious disease physician	62	56.4			
Acute physician	26	23.6			
Nurse	7	6.4			
Clinical microbiologist	6	5.4			
Consultant paediatrician	3	2.7			
Clinical pharmacist	1	0.9			
Other^b^	5	4.5			
OPAT programme falls under the responsibility of the AMS team (*n* = 147)					
Yes	87	59.2	76	87.4	0.009
No	60	40.8	42	70	
Type of OPAT model of care (*n* = 147)					
S-OPAT	6	4.1	5	83.3	0.765
C-OPAT	32	21.8	27	84.4	
H-OPAT	36	24.5	26	72.2	
S-OPAT and C-OPAT	6	4.1	5	83.3	
H-OPAT and C-OPAT	13	8.8	11	84.6	
H-OPAT and S-OPAT	25	17.0	22	88	
All models	29	19.7	22	75.9	
Mandatory ID physician consultation before discharging a patient (*n* = 147)					
Yes	104	70.7	86	82.7	0.251
No	43	29.3	32	74.4	
Patients have been discharged with OPAT without seeing any member of the ID team (*n* = 147)					
Yes	52	35.4	44	84.6	0.328
No	95	64.6	74	77.9	
Monitoring patients for antimicrobial toxicity (*n* = 147)					
Yes	126	85.7	109	86.5	<0.001
No	21	14.3	9	42.9	
Frequency of patients’ antimicrobial toxicity monitoring (*n* = 126)					
Rarely	4	3.2	4	100	0.755
Once a week	50	39.7	43	86	
Twice a week	9	7.1	9	100	
It depends on the antimicrobial	63	50.0	53	84.1	
Systematic method of tracking patient monitoring (*n* = 126)					
Yes	103	81.7	94	91.3	0.003
No	23	18.3	15	65.2	
Systematic method of ensuring patient follow-up (*n* = 147)					
Yes	123	83.7	105	85.4	0.001
No	24	16.3	13	54.2	
Training programme for patients and carers who choose to self-administer (*n* = 147)					
Yes	70	47.6	61	87.1	0.046
No	77	52.4	57	74	
Audit of OPAT programme (*n* = 147)					
Yes	58	39.5	52	89.7	0.021
No	89	60.5	66	74.2	
Outcome measure for evaluating the OPAT programme (*n* = 58)					
Completion of OPAT as planned	51	87.9	47	92.2	0.008
Complication	40	69.0	36	90	0.070
Side effects of antimicrobials	38	65.5	35	92.1	0.033
Readmission rate	37	63.8	34	91.9	0.040
Clinical cure	36	62.1	33	91.7	0.048
Patient satisfaction	34	58.6	32	94.1	0.021
Laboratory monitoring	25	43.1	22	88	0.410
Emergency department visit during OPAT	23	39.7	21	91.3	0.251
Patient survival	19	32.8	17	89.5	0.367
Other^c^	6	10.3	6	100	0.599

AMS, antimicrobial stewardship; C-OPAT, clinic or infusion centre administration; H-OPAT, physician- or nurse-administered OPAT at the patient's home; NA, not applicable (association not computed because these questions were answered only by facilities with a formal OPAT service structure); OPAT, outpatient parenteral antimicrobial therapy; S-OPAT, self- or carer-administered OPAT at the patient's home.

^a^Pharmacist, pharmacy technician, clinical support worker, vascular access team, family doctor, hospital-at-home physician, geriatric specialist, general practitioner, social worker, nurse, physiotherapist and medical assistant.

^b^Medical specialist, internist and general practitioner.

^c^Patient demographics, rate of bacteraemia, history of multidrug-resistant infection, staffing, elastomeric pump ordering, adherence to evidence-based guidelines, reasons for decline of OPAT service and days saved.

### Barriers to OPAT implementation

**Figure 1.**
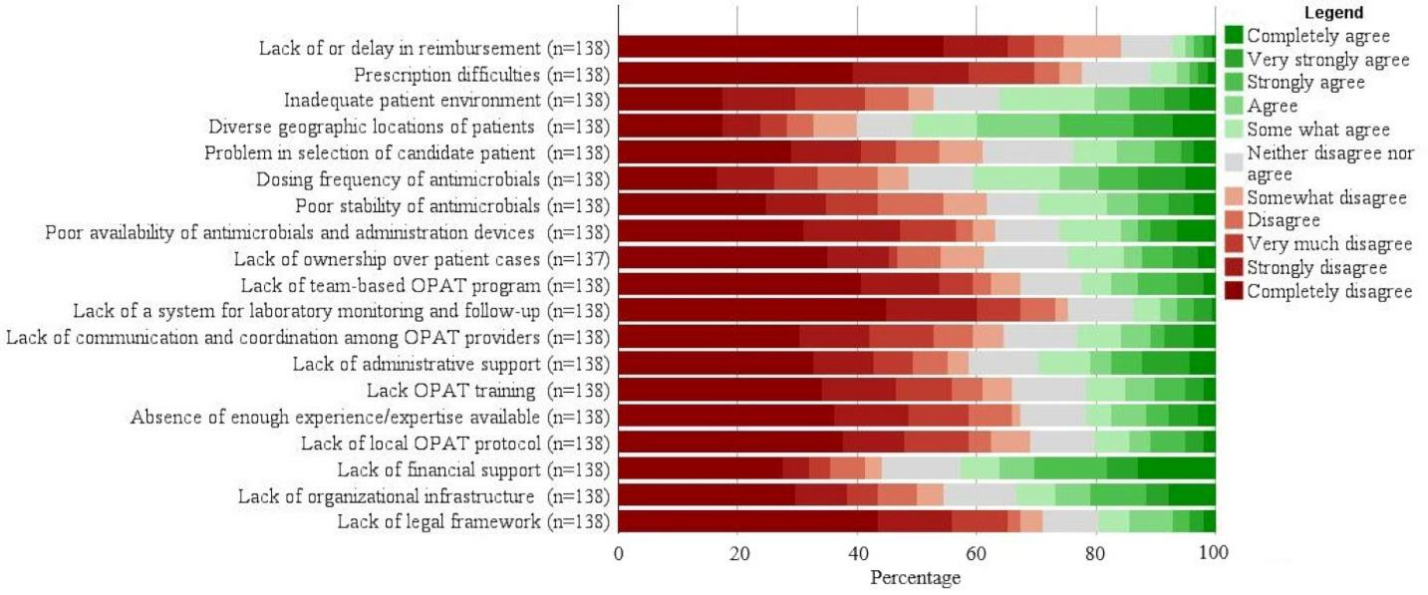
Barriers to OPAT service implementation.

Figure 1 presents the reported barriers to the implementation of OPAT. The most frequently experienced challenges include the diverse geographic distribution of patients (50.7%), lack of financial support (42.7%) and the dosing frequency of antimicrobials (40.6%). Other barriers include inadequate patient home environments (36.1%), lack of organizational infrastructure (33.1%), insufficient administrative support (29.7%), poor antimicrobial stability (29.7%), limited availability of antimicrobials and administration devices (26.0%), difficulties in selecting appropriate patient candidates (23.8%) and poor communication and coordination among OPAT providers (23.2%). Further details on these barriers are available in Table S1.

### Facilitators to OPAT implementation

**Figure 2.**
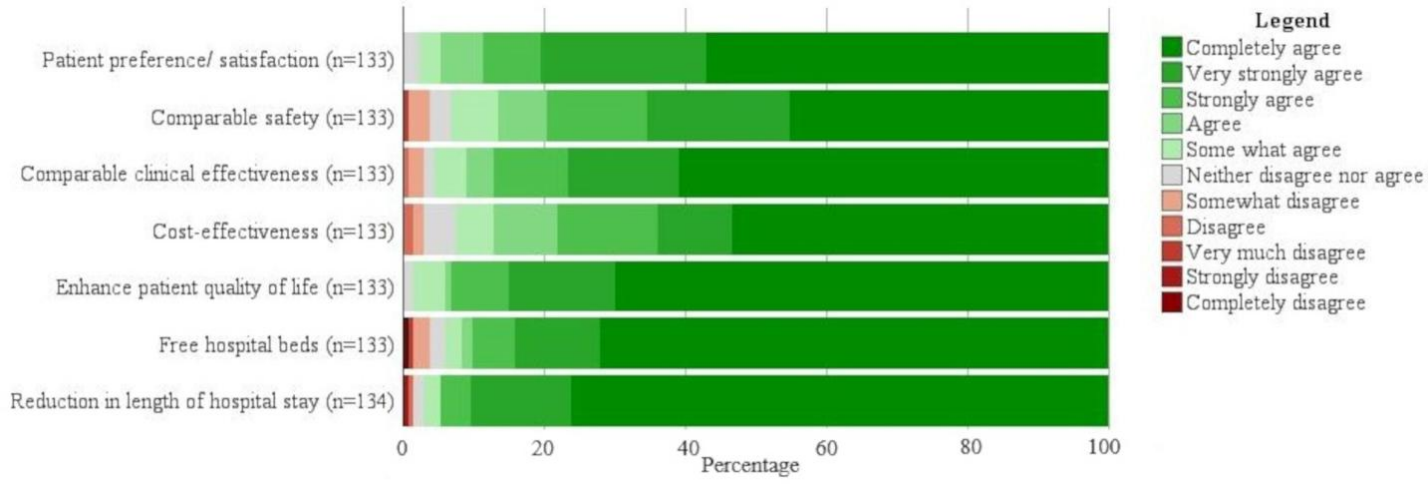
Facilitators to OPAT services implementation.

Figure 2 presents the reported facilitators of OPAT implementation. Key factors supporting successful implementation include improved patient quality of life (98.5%), high patient satisfaction (97.7%), reduction in hospital length of stay (97.0%), increased availability of hospital beds (94.0%), clinical efficacy (95.5%), safety (93.2%) and cost-effectiveness (92.5%) of the OPAT programme. Further details on these facilitators are provided in Table S2.

A subgroup analysis was conducted on the implementation of OPAT services in Spain (Tables S3–S5), the UK (Tables S6–S8), Australia (Tables S9–S11), Turkey (Tables S12–S14) and Malaysia (Tables S15–S17). Overall, the implementation characteristics in these countries were generally consistent with the findings of the main analysis. However, notable differences were observed. In Turkey, all OPAT services were implemented in urban areas. In Malaysia, the majority of OPAT services were implemented in non-teaching hospitals, each serving only 1–5 patients per month.

The majority of health facilities implement OPAT through a formal OPAT structure. In facilities with a formal structure, an ID physician, a clinical pharmacist and a specialist nurse are commonly included as members of the OPAT team in Spain, the UK, Australia and Malaysia. Notably, all participating facilities from Turkey and Malaysia implemented OPAT under a designated formal team lead. Except in Spain, most OPAT programmes in participating facilities are implemented as part of an AMS team. In Spain, nearly half of the facilities (49.0%) reported implementing physician- or nurse-administered OPAT at the patient’s home (H-OPAT), whereas all facilities in Malaysia exclusively provided OPAT at clinics or infusion centres (C-OPAT). Although in most countries an ID physician consultation before patient discharge is mandatory, a significant number of centres discharge patients without an ID physician consultation (Table S6). Training for patients and carers choosing self-administration is provided in the majority of facilities in the UK (76.7%) and Malaysia (81.3%). Additionally, OPAT programme audits are implemented in 33 facilities (76.7%) in the UK.

The relationship between mandatory ID consultation and the integration of OPAT programmes within an AMS programme across OPAT settings was assessed, and no significant association was found (Table S18).

## Discussion

This survey assessed the implementation of OPAT services and identified barriers and facilitators influencing their delivery. The most frequently reported barriers include the wide geographic distribution of patients, lack of financial support, frequent antimicrobial dosing requirements and inadequate home environments. In contrast, facilitators include hospital bed savings, clinical safety and efficacy, cost-effectiveness and high patient satisfaction with outpatient care. Self- or carer-administered OPAT at the patient's home (S-OPAT) was a less commonly practised mode of administration. The majority of facilities delivered OPAT through a formal service structure that involved a multidisciplinary team, with clinical leadership provided by an ID physician in more than half of the programmes. The formal OPAT programmes are characterized by integration within an AMS team, regular audits, structured patient monitoring and follow-up.

This study identified several challenges in OPAT implementation, with the diverse geographic distribution of patients emerging as a significant barrier. Providing consistent and timely care is particularly difficult in rural or remote areas, where access to healthcare services is often limited. For example, a study conducted in rural France reported limited OPAT implementation due to such geographic constraints.^16^ The integration of telemedicine offers a promising solution by enabling remote monitoring and consultation, thereby reducing travel burdens and improving access for patients in underserved regions or those with mobility impairments.^29^ Additionally, S-OPAT presents an alternative model, allowing patients residing outside catchment areas to receive initial training in hospital settings and attend follow-up visits less frequently.^3,30^

In the current study, fewer than half of the surveyed health facilities had implemented S-OPAT. In comparison, a survey across 28 European countries reported S-OPAT implementation in 10 countries (58.8%), with Switzerland and the UK considering it standard practice.^15^ In this study, 36 facilities (83.7%) in the UK provided S-OPAT. In the Netherlands, only 30% of hospitals were found to offer this modality,^19^ while implementation rates are higher (94.1%) in the UK.^17^ Given that S-OPAT is considered safe, effective, convenient and cost-efficient when patients receive appropriate training, its broader implementation is important to meet the growing healthcare demands and patient-centred care.^31,32^

Antimicrobial dosing frequency and stability are critical determinants of OPAT feasibility. Antimicrobials requiring more frequent administration are impractical for H-OPAT and C-OPAT models and are not preferable for the S-OPAT model either, despite being feasible.^30^ While continuous infusion via portable pumps may offer a solution, their use is limited by the stability of many antimicrobials.^33^ The stability of antimicrobials under varying storage and infusion conditions may compromise therapeutic efficacy and increase the risk of toxicity from degradation products.^34^ A 2025 survey identified several antimicrobials for which additional stability data are needed to support their use in OPAT and revealed substantial variation in acceptable degradation thresholds, highlighting the absence of a universally accepted standard.^8^ These findings emphasize the need for harmonized antimicrobial stability data and dosing guidelines. Standardizing national protocols, while allowing for local adaptation, is essential to ensure the safe, consistent and scalable delivery of OPAT across diverse healthcare settings.

Lack of adequate financial, administrative and infrastructure support remains a barrier to effective OPAT implementation, as consistently reported in previous studies.^15,19,24^ Financial limitations restrict the capacity of healthcare institutions to sustain existing OPAT programmes or establish new ones, particularly in resource-limited settings.^35^ Inadequate infrastructure, including unsuitable home environments for patients, persists even in high-income countries, impeding the development of standardized clinical pathways, coordinated care models and reliable follow-up systems.^15,36,37^ Additionally, a lack of administrative support hinders logistical coordination, delays service delivery and places additional strain on clinical teams.^38^

Despite the health system-level funding and administrative support constraints, ironically evidence from key health economic analyses suggests that OPAT is a cost-effective approach that optimizes the use of healthcare resources and offers huge cost savings for the healthcare system in the long run.^39^ Thus, its implementation is likely to continue to expand driven by economic pressures on healthcare systems^40^ and a growing emphasis on delivering care closer to patients’ homes.^41^ Key facilitators of OPAT implementation include its demonstrated clinical effectiveness,^6^ hospital bed savings, cost-efficiency^12,42^ and high levels of patient satisfaction.^43,44^

Previous global^8^ and continental studies^15^ have compared OPAT implementation across countries, revealing substantial variation in practice. These differences are attributed to differences in adopted guidelines^13^ and varying levels of adherence to practise recommendations,^24^ leading to diverse organization of OPAT services worldwide. For instance, a 2017 survey across 17 Asian countries highlighted significant inconsistencies: comprehensive OPAT services were reported by only 11 institutions in 6 countries; infusion services were available in 36 institutions across 9 countries and outpatient parenteral antimicrobials were administered via individual clinics in 47 inpatient institutions across 10 countries, as well as in 3 outpatient institutions in 3 countries.^23^ Similarly, a 2022 European survey found that 14.3% of countries lacked structured OPAT services, with care instead delivered through *ad hoc* arrangements.^15^

Compared with previous studies, the implementation of OPAT through a formal service structure appears to be higher in the current study. In Europe, a formal OPAT team structure has been reported in only six countries (35.3%).^15^ In the Netherlands, 22 hospitals (40.0%) had a team-based OPAT programme.^19^ An international survey found that 42% of respondents provided formal OPAT services through a designated team,^24^ while in the USA, 36% of ID physicians reported having a formal OPAT programme through a dedicated team.^20^ The UK OPAT guidelines recommend that the multidisciplinary OPAT team be led by a clinician or an infection specialist with experience in OPAT.^9^ However, in the current study, 6.8% of health facilities reported having no designated clinical lead, highlighting the need for clearly defined leadership roles to ensure high-quality care.

The implementation of a formal OPAT structure was found to be characterized by integration within an AMS team, regular audits, structured patient monitoring and follow-up. These findings are consistent with previous studies.^19,21,24^ In 59.2% of the surveyed health facilities, the OPAT programme was overseen by the AMS team. This finding is lower than that of the Dutch national study, where 77.3% of hospitals placed OPAT under AMS oversight.^19^ A European study found that in nine countries (52.9%), OPAT team members were part of the AMS team and in four countries (23.5%), OPAT services were formally integrated into AMS programmes.^15^ Integration with AMS has been shown to reduce inappropriate antimicrobial use and improve patient outcomes.^45^ This has also facilitated the emergence of Complex Outpatient Antimicrobial Therapy (COpAT) programme whereby a switch from intravenous to oral antimicrobials, where clinically appropriate, is increasingly considered within the operational structures of the traditional OPAT service.^46^ COpAT broadens the scope of the OPAT programme by shifting the care model from parenteral to a more flexible, patient-centred approach that combines the benefits of traditional OPAT with the advantages of oral antimicrobials. Evidence shows that COpAT is non-inferior to parenteral therapy in the management of severe infections.^47–50^ It enhances patient experience by removing the burden of daily infusions and nursing time,^48^ thereby lowering the risk of catheter-related bloodstream infections.^51^ However, it requires careful patient selection.

OPAT guidelines recommend that an ID expert review be conducted before initiating OPAT.^5^ In the current study, 70.7% of health facilities implemented mandatory ID consultation before starting OPAT. The finding is higher than those reported in the USA, where only 37% of adult OPAT programmes^20^ and 15% of paediatric sites^22^ mandated such consultation. It also exceeds findings from an international survey (53.1%)^24^ and a Dutch national study (31.8%).^19^ The involvement of ID physicians in OPAT care has been associated with improved patient outcomes, substantial cost savings^52^ and a reduction in emergency department visits.^53^ Additionally, ID oversight contributes to optimized antimicrobial therapy and a reduction in inappropriate antimicrobial use.^54,55^ In settings lacking on-site ID consultation, a telehealth initiative has demonstrated success in extending ID expertise to underserved community hospitals.^56^

Previous studies have demonstrated that facilities with formal OPAT programmes are more likely to conduct follow-up appointments and laboratory monitoring compared with those without such structures.^19,21,24^ This aligns with the findings of the current survey, in which 81.7% and 83.7% of facilities reported implementing systematic patient monitoring and follow-up, respectively. Administering parenteral antimicrobials in unmonitored settings increases the risk of adverse events and hospital readmissions. Adverse events have been reported in up to 18% of patients discharged on OPAT.^57^ Current OPAT guidelines recommend routine laboratory testing for all patients receiving OPAT to monitor for potential complications.^5,30^ However, there remains limited evidence to guide the optimal frequency of laboratory testing. In addition, the relevance of laboratory tests commonly used for OPAT follow-up is not clearly known.^58,59^ These findings highlight the need for a national programme to ensure systematic follow-up and monitoring of OPAT patients.^18^

Although most health facilities have implemented a formal OPAT programme, fewer than half have conducted evaluations of its implementation. Effective delivery of OPAT services requires structured quality improvement initiatives and routine audits.^18^ The UK's good practice recommendations emphasize the need for standardized outcome measures for OPAT.^5^ However, inconsistencies persist in the quality metrics used to assess OPAT outcomes. A 2025 survey of OPAT team members in the USA identified readmission rates, adverse reactions to antimicrobials and clinical outcomes as the most highly valued indicators for programme evaluation.^60^ Despite this, our survey found limited and inconsistent use of these metrics across facilities in assessing OPAT programme performance.

Regular assessment of OPAT outcomes against national standards enables ongoing improvements. To standardize the OPAT programme and implement best practices, initiatives such as expanding telemedicine options for patients in remote areas and those with mobility difficulties, integrating health technologies into OPAT, allocating adequate staff and resources, investing in infrastructure and utilizing community-based care models are advantageous.

The strength of this study lies in the development of the data collection tool, which was based on a thorough review of the literature and the CFIR.^25^ Each OPAT centre contributed a single, team-based response, enhancing the reliability of the data. Compared with previous studies, this survey achieved a higher number of responses from health facilities providing OPAT services, likely due to its distribution through professional associations and networks, the use of a concise electronic questionnaire and the implementation of follow-up reminders. However, the study is not without limitations. Responses may be subject to recall bias, although this was mitigated by recording team responses. The absence of precise data on the total number of OPAT centres limits the ability to assess representativeness, and notably, response rates from Spain, Australia and the UK appeared relatively low. The survey did not differentiate barriers across specific OPAT care models, nor did it explore implementation challenges in depth. Further research is warranted to examine care model–specific factors, including qualitative studies exploring barriers to OPAT implementation.

In conclusion, this study demonstrates that most health facilities implement OPAT services through a formal structure, though variations in practice remain. The implementation of a formal OPAT programme is associated with improvements in several aspects of care. However, multiple barriers continue to hinder the effective delivery of OPAT services. Addressing these challenges will require targeted efforts to overcome organizational barriers and to enhance institutional support for OPAT services.

## Supplementary Material

dlag095_Supplementary_Data

## Data statement

The data underlying this study are available in the article and Supplementary data.

## Supplementary data

Tables S1–S18 are available as Supplementary data at *JAC-AMR* Online.

## References

[1] Rucker RW, Harrison GM. Outpatient intravenous medications in the management of cystic fibrosis. Pediatrics 1974; 54: 358–60. 10.1542/peds.54.3.358 4213282

[2] Howden BP, Grayson ML. 5: Hospital-in-the-home treatment of infectious diseases. Med J Aust 2002; 176: 440–5. 10.5694/j.1326-5377.2002.tb04450.x 12056999

[3] Tice AD, Rehm SJ, Dalovisio JR et al Practice guidelines for outpatient parenteral antimicrobial therapy. Clin Infect Dis 2004; 38: 1651–71. 10.1086/420939 15227610

[4] Mitchell E, Murray CC, Meads D et al Clinical and cost-effectiveness, safety and acceptability of community intravenous antibiotic service models: CIVAS systematic review. BMJ Open 2017; 7: e013560. 10.1136/bmjopen-2016-013560 PMC5775457 28428184

[5] Chapman AL, Patel S, Horner C et al Updated good practice recommendations for outpatient parenteral antimicrobial therapy (OPAT) in adults and children in the UK. JAC Antimicrob Resist 2019; 1: dlz026. 10.1093/jacamr/dlz026 34222901 PMC8209972

[6] Mohammed SA, Roberts JA, Cotta MO et al Safety and efficacy of outpatient parenteral antimicrobial therapy: a systematic review and meta-analysis of randomized clinical trials. Int J Antimicrob Agents 2024; 64: 107263. 10.1016/j.ijantimicag.2024.107263 38960209

[7] Erba A, Beuret M, Daly ML et al OPAT in Switzerland: single-center experience of a model to treat complicated infections. Infection 2020; 48: 231–40. 10.1007/s15010-019-01381-8 31828605

[8] Wolie ZT, Roberts JA, López-Cortés LE et al Current practices in outpatient parenteral antimicrobial therapy programmes: an international multi-centre survey. JAC Antimicrob Resist 2025; 7: dlaf075. 10.1093/jacamr/dlaf075 40433448 PMC12107060

[9] Chapman AL, Seaton RA, Cooper MA et al Good practice recommendations for outpatient parenteral antimicrobial therapy (OPAT) in adults in the UK: a consensus statement. J Antimicrob Chemother 2012; 67: 1053–62. 10.1093/jac/dks003 22298347

[10] Berrevoets MAH, Oerlemans AJM, Tromp M et al Quality of outpatient parenteral antimicrobial therapy (OPAT) care from the patient's perspective: a qualitative study. BMJ Open 2018; 8: e024564. 10.1136/bmjopen-2018-024564 PMC6252647 30420352

[11] Carter B, Fisher-Smith D, Porter D et al Being ‘at-home’ on outpatient parenteral antimicrobial therapy (OPAT): a qualitative study of parents’ experiences of paediatric OPAT. Arch Dis Child 2020; 105: 276–81. 10.1136/archdischild-2019-317629 31558443 PMC7041500

[12] Mohammed SA, Roberts JA, Mirón-Rubio M et al Quantifying cost savings from outpatient parenteral antimicrobial therapy programme: a systematic review and meta-analysis. JAC Antimicrob Resist 2025; 7: dlaf049. 10.1093/jacamr/dlaf049 40201538 PMC11976721

[13] Esposito S, Noviello S, Leone S et al Outpatient parenteral antibiotic therapy (OPAT) in different countries: a comparison. Int J Antimicrob agents 2004; 24: 473–8. 10.1016/j.ijantimicag.2004.06.004 15519480

[14] Nathwani D, Zambrowski JJ. Advisory group on Home-based and Outpatient Care (AdHOC): an international consensus statement on non-inpatient parenteral therapy. Clin Microbiol Infect 2000; 6: 464–76. 10.1046/j.1469-0691.2000.00113.x 11168180

[15] Emilie C, de Nocker P, Saïdani N et al Survey of delivery of parenteral antimicrobials in non-inpatient settings across Europe. Int J Antimicrob Agents 2022; 59: 106559. 10.1016/j.ijantimicag.2022.106559 35227827

[16] Triffault-Fillit C, Ferry T, Perpoint T et al Outpatient parenteral antibiotic therapy: evaluation of practices and limits of use in rural areas in France. Med Mal Infects 2018; 48: 130–5. 10.1016/j.medmal.2017.09.008 29050864

[17] Durojaiye OC, Cartwright K, Ntziora F. Outpatient parenteral antimicrobial therapy (OPAT) in the UK: a cross-sectional survey of acute hospital trusts and health boards. Diagn Microbiol Infect Dis 2019; 93: 58–62. 10.1016/j.diagmicrobio.2018.07.013 30098851

[18] Muldoon EG, Allison GM, Gallagher D et al Outpatient parenteral antimicrobial therapy (OPAT) in the Republic of Ireland: results of a national survey. Eur J Clin Microbiol Infect Dis 2013; 32: 1465–70. 10.1007/s10096-013-1899-4 23728737 PMC3973129

[19] Stoorvogel HH, Hulscher MEJL, Wertheim HFL et al Current practices and opportunities for outpatient parenteral antimicrobial therapy in hospitals: a National Cross-Sectional Survey. Antibiotics 2022; 11: 1343. 10.390/antibiotics11101343 36290001 PMC9598700

[20] Hamad Y, Lane MA, Beekmann SE et al Perspectives of United States-based infectious diseases physicians on outpatient parenteral antimicrobial therapy practice. Open Forum Infect 2019; 6: ofz363. 10.1093/ofid/ofz363 PMC6765349 31429872

[21] Muldoon EG, Switkowski K, Tice A et al A national survey of infectious disease practitioners on their use of outpatient parenteral antimicrobial therapy (OPAT). Infect Dis 2015; 47: 39–45. 10.3109/00365548.2014.967290 25415655

[22] Vaz LE, Felder KK, Newland JG et al National survey of outpatient parenteral antibiotic therapy practices. J Pediatr Infect Dis Soc 2022; 11: 115–8. 10.1093/jpids/piab127 34939654

[23] Fisher D, Michaels J, Hase R et al Outpatient parenteral antibiotic therapy (OPAT) in Asia: missing an opportunity. J Antimicrob Chemother 2017; 72: 1221–6. 10.1093/jac/dkw551 28077673

[24] Hassanzai M, Adanç F, Koch B et al Best practices, implementation and challenges of outpatient parenteral antimicrobial therapy: results of a worldwide survey among healthcare providers. Ther Adv Infect Dis 2023; 10: 20499361231214901. 10.1177/20499361231214901 38127471 PMC10722947

[25] Mohammed SA, Cotta MO, Assefa GM et al Barriers and facilitators for the implementation and expansion of outpatient parenteral antimicrobial therapy: systematic review. J Hospital Infect 2024; 147: 1–16. 10.1016/j.jhin.2024.02.00 38423135

[26] Damschroder LJ, Reardon CM, Widerquist MAO et al The updated consolidated framework for implementation research based on user feedback. Implementat Sci 2022; 17: 75. 10.1186/s13012-022-01245-0 PMC9617234 36309746

[27] Chary A, Tice AD, Martinelli LP et al Experience of infectious diseases consultants with outpatient parenteral antimicrobial therapy: results of an emerging infections network survey. Clin Infect Dis 2006; 43: 1290–5. 10.1086/508456 17051494

[28] Lane MA, Marschall J, Beekmann SE et al Outpatient parenteral antimicrobial therapy practices among adult infectious disease physicians. Infect Control HospEpidemiol 2014; 35: 839–44. 10.1086/676859 PMC4180108 24915212

[29] Durojaiye OC, Jibril I, Kritsotakis EI. Effectiveness of telemedicine in outpatient parenteral antimicrobial therapy (Tele-OPAT): a systematic review. J Telemed Telecare 2024; 30: 1230–37. 10.1177/1357633X221131842 36221964

[30] Norris AH, Shrestha NK, Allison GM et al 2018 Infectious Diseases Society of America clinical practice guideline for the management of outpatient parenteral antimicrobial therapy. Clin Infect Dis 2019; 68: e1–35. 10.1093/cid/ciy745 30423035

[31] Subedi S, Looke DF, McDougall DA et al Supervised self-administration of outpatient parenteral antibiotic therapy: a report from a large tertiary hospital in Australia. Int J Infect Dis 2015; 30: 161–5. 10.1016/j.ijid.2014.11.021 25603999

[32] Mujal A, Sola J, Hernandez M et al Safety and effectiveness of outpatient parenteral antimicrobial therapy in older people. J Antimicrob Chemother 2016; 71: 1402–7. 10.1093/jac/dkv478 26832749

[33] Van Abel AL, Childs-Kean LM, Jensen KL et al A review of evidence, antimicrobial stability, and feasibility considerations for OPAT continuous infusion. Ther Adv Infect Dis 2023; 10: 20499361231191877. 10.1177/20499361231191877 37636216 PMC10451047

[34] Voumard R, Van Neyghem N, Cochet C et al Antibiotic stability related to temperature variations in elastomeric pumps used for outpatient parenteral antimicrobial therapy (OPAT). J Antimicrob Chemother 2017; 72: 1462–5. 10.1093/jac/dkw582 28158637

[35] Durojaiye OC, Fiori C, Cartwright K. Delivery of Outpatient Parenteral Antimicrobial Therapy (OPAT) in an ever-changing National Health Service (UK): benefits, barriers, and opportunities. Antibiotics 2025; 14: 451. 10.3390/antibiotics14050451 40426518 PMC12108282

[36] Yan M, Lam PW, Andany N et al Assessing the utilization and impact of a newly established outpatient parenteral antimicrobial therapy (OPAT) program. J Assoc Med Microbiol Infect Dis Can 2020; 5: 70–6. 10.3138/jammi.2019-0018 36338181 PMC9602881

[37] Keller SC, Cosgrove SE, Kohut M et al Hazards from physical attributes of the home environment among patients on outpatient parenteral antimicrobial therapy. Am J Infect Control 2019; 47: 425–30. 10.1016/j.ajic.2018.09.020 30471975 PMC6893846

[38] Agnihotri G, Gross AE, Seok M et al Decreased hospital readmissions after programmatic strengthening of an outpatient parenteral antimicrobial therapy (OPAT) program. Antimicrob Steward Healthc Epidemiol 2023; 3: e33. 10.1017/ash.2022.330 36865701 PMC9972539

[39] Dimitrova M, Gilchrist M, Seaton R. Outpatient parenteral antimicrobial therapy (OPAT) versus inpatient care in the UK: a health economic assessment for six key diagnoses. BMJ Open 2021; 11: e049733. 10.1136/bmjopen-2021-049733 PMC8479950 34588251

[40] Chen X, Geng S, Zhu Y et al Impact of infection on healthcare costs and clinical outcomes in elderly hospitalized patients with multimorbidity. Heliyon 2024; 10: e31560. 10.1016/j.heliyon.2024.e31560 38826722 PMC11141361

[41] Prakash B . Patient satisfaction. J Cutan Aesthet Surg 2010; 3: 151–5. 10.4103/0974-2077.74491 21430827 PMC3047732

[42] Psaltikidis EM, Silva E, Bustorff-Silva JM et al Economic evaluation of outpatient parenteral antimicrobial therapy: a systematic review. Expert Rev Pharmacoeconomics Outcomes Res 2017; 17: 355–75. 10.1080/14737167.2017.1360767 28776441

[43] Peter S, Oberröhrmann C, Pfaff H et al Exploring patients’ perspectives: a mixed methods study on Outpatient Parenteral Antimicrobial Therapy (OPAT) experiences. BMC Health Serv Res 2024; 24: 544. 10.1186/s12913-024-11017-9 38685017 PMC11057129

[44] Carter B, Fisher-Smith D, Porter D et al Paediatric Outpatient Parenteral Antimicrobial Therapy (OPAT): an e-survey of the experiences of parents and clinicians. PLoS One 2021; 16: e0249514. 10.1371/journal.pone.0249514 33798226 PMC8018658

[45] Huynh J, Hodgson KA, Boyce S et al Impact of expanding a paediatric OPAT programme with an antimicrobial stewardship intervention. Arch Dis Child 2020; 105: 1220–8. 10.1136/archdischild-2019-318091 32381516

[46] Seaton R, Ritchie N, Robb F et al From ‘OPAT’ to ‘COpAT’: implications of the OVIVA study for ambulatory management of bone and joint infection. J Antimicrob Chemother 2019; 74: 2119–21. 10.1093/jac/dkz122 30989175

[47] Stets R, Popescu M, Gonong JR et al Omadacycline for community-acquired bacterial pneumonia. N Engl J Med 2019; 380: 517–27. 10.1056/NEJMoa180020 30726692

[48] Seaton RA, Bell E, Gourlay Y et al Nurse-led management of uncomplicated cellulitis in the community: evaluation of a protocol incorporating intravenous ceftriaxone. J Antimicrob Chemother 2005; 55: 764–7. 10.1093/jac/dki092 15772137

[49] Tamma PD, Conley AT, Cosgrove SE et al Association of 30-day mortality with oral step-down vs continued intravenous therapy in patients hospitalized with Enterobacteriaceae bacteremia. JAMA Intern Med 2019; 179: 316–23. 10.1001/jamainternmed.2018.6226 30667477 PMC6439703

[50] Li H-K, Rombach I, Zambellas R et al Oral versus intravenous antibiotics for bone and joint infection. N Engl J Med 2019; 380: 425–36. 10.1056/NEJMoa1710926 30699315 PMC6522347

[51] Dryden M, Saeed K, Townsend R et al Antibiotic stewardship and early discharge from hospital: impact of a structured approach to antimicrobial management. J Antimicrob Chemother 2012; 67: 2289–96. 10.1093/jac/dks193 22623629

[52] Conant MM, Erdman SM, Osterholzer D. Mandatory infectious diseases approval of outpatient parenteral antimicrobial therapy (OPAT): clinical and economic outcomes of averted cases. J Antimicrob Chemother 2014; 69: 1695–700. 10.1093/jac/dku015 24532684

[53] Shah A, Petrak R, Fliegelman R et al Infectious diseases specialty intervention is associated with better outcomes among privately insured individuals receiving outpatient parenteral antimicrobial therapy. Clin Infect Dis 2019; 68: 1160–5. 10.1093/cid/ciy674 30247512

[54] Sharma R, Loomis W, Brown RB. Impact of mandatory inpatient infectious disease consultation on outpatient parenteral antibiotic therapy. Am J Med Sci 2005; 330: 60–4. 10.1097/00000441-200508000-00002 16103785

[55] Shrestha NK, Bhaskaran A, Scalera NM et al Contribution of infectious disease consultation toward the care of inpatients being considered for community-based parenteral anti-infective therapy. J Hospital Med 2012; 7: 365–9. 10.1002/jhm.1902 22315151

[56] Davis MR, Shah N, Maurer SM et al P-1890. Implementation and outcomes of a fully remote tele-ID and tele-OPAT program at a regional medical center. Open Forum Infect Dis 2025; 12(Suppl 1): ofae631. 2051. 10.1093/ofid/ofae631.2051

[57] Keller SC, Williams D, Gavgani M et al Rates of and risk factors for adverse drug events in outpatient parenteral antimicrobial therapy. Clin Infect Dis 2018; 66: 11–9. 10.1093/cid/cix733 29020202 PMC5848264

[58] Frisby J, Ali N, Niemotka S et al Usefulness of routine laboratory tests for follow up of patients receiving outpatient parenteral antimicrobial therapy run by infectious diseases fellows. Antibiotics 2023; 12: 330. 10.3390/antibiotics12020330 36830241 PMC9952172

[59] Stoorvogel HH, van Egmond M, Wertheim HF et al Occurrence and predictors of laboratory abnormalities during outpatient parenteral antimicrobial therapy—A multicenter cohort study to inform laboratory test monitoring. J Infect 2024; 89: 106301. 10.1016/j.jinf.2024.106301 39357569

[60] Makadia J, Streifel AC, Varley CD. Outpatient parenteral antimicrobial therapy outcomes metrics assessment survey. Open Forum Infect Dis 2025; 12: ofaf283. 10.1093/ofid/ofaf283 40438299 PMC12117654

