# Supplementary material for: Challenges and drivers in the implementation of current practices in outpatient parenteral antimicrobial therapy: an international survey
Source: JAC Antimicrob Resist. 2026 May 26;8(3):dlag095. doi: 10.1093/jacamr/dlag095 (PMC13201090; doi:10.1093/jacamr/dlag095)
Supplement: dlag095_Supplementary_Data [file dlag095_supplementary_data.docx]

**Supplementary information A**

**Data collection tool**

We would like to have one response per OPAT centre. Please submit one reply for your OPAT program, after discussing it with OPAT team members.

**Part 1: Characteristics of the OPAT program**

1. Name of your OPAT centreClick or tap here to enter text.
2. Mail address Click or tap here to enter text.
3. In which country is your OPAT program located?Click or tap here to enter text.
4. How would you best describe the location of your practice?

Urban

Suburban

Rural

1. Which best describes your OPAT setting?

University teaching hospital

Community teaching hospital

Non-teaching hospital

Veteran administration

Other, specify Click or tap here to enter text.

1. What type(s) of OPAT are you practicing?

Adult OPAT only

Paediatric OPAT only

A combination of adult and paediatric OPAT

1. On average, how many patients would your OPAT program manage monthly?

1-5 patients per month

6-15 patients per month

16-25 patients per month

26-50 patients per month

More than 50 patients per month

**Part 2: OPAT implementation**

**OPAT team and structure**

1. Does your facility have a formal OPAT service structure*?

Yes

No

* A formal OPAT service structure is the presence of care coordination with clear clinical and managerial accountabilities of health professionals involved in the OPAT care pathway.

If no, skip to question number 11

1. In your OPAT service, who are the members of your OPAT team? (Check all that apply)

Infectious diseases physician

Clinical microbiologist

Acute physician

Consultant paediatrician

Advanced nurse practitioner

Specialist nurse

Community nurse

Clinical pharmacist

Administrative assistant

IT specialist

Others, specify Click or tap here to enter text.

1. Who is the clinical leader of your OPAT team?

No formal lead

Infectious diseases physician

Clinical microbiologist

Acute physician

Consultant paediatrician

Nurse

Clinical pharmacist

Others, specify Click or tap here to enter text.

1. Does your OPAT program fall under the responsibility of the antimicrobial stewardship team?

Yes

No

1. What type of OPAT model of care you are practicing? (Check all that apply)

Self, or carer-administration of OPAT at patients’ homes (S-OPAT)

Delivered by visiting health professionals at the patient’s home (H-OPAT)

Delivered in a clinic or infusion centre

Other, specify Click or tap here to enter text.

**Patient selection**

1. Is an ID physician consultation mandatory before discharging a patient with OPAT at your practice site?

Yes

No

1. Has there been any instance where patients have been discharged with OPAT without seeing any member of the ID team before discharge?

Yes

No

**Patient monitoring and follow-up**

1. Does your OPAT program monitor patients for antimicrobial toxicity?

Yes

No

If no, skip to question number 18

1. How frequently do you monitor patients for antimicrobial toxicity?

Rarely

Every other week

Once a week

Twice a week

It depends on the antimicrobial

1. Is there any systematic method of tracking patient monitoring, i.e. are you able to know which patients’ laboratory results are overdue?

Yes

No

1. Is there a systematic method of ensuring patient follow-up in your practice?

Yes

No

**OPAT education and training**

1. Is there a structured training program for patients and caregivers who choose to self-administer OPAT?

Yes

No

**OPAT-related outcome measures**

1. Have you ever performed an audit of your OPAT program?

Yes

No

If no, skip to question part 3

1. Which outcome measures were used to monitor your OPAT program? (Check all that apply)

Completion of OPAT as planned

Side effects of antimicrobials

Clinical cure

Readmission rate

Emergency department visit during OPAT

Complication

Patient satisfaction

Patient survival

Laboratory monitoring

Other, specify Click or tap here to enter text.

**Part 3: Barriers and facilitators to the implementation of the OPAT program**

1. **Barriers to the OPAT program**

Why are OPAT services NOT easily available to all eligible patients in your health facility? Because….

Please rate your level of agreement on a scale from 0 (completely disagree) to 10 (completely agree).

|  | 0 | 1 | 2 | 3 | 4 | 5 | 6 | 7 | 8 | 9 | 10 |
| --- | --- | --- | --- | --- | --- | --- | --- | --- | --- | --- | --- |
| Lack of legal framework |  |  |  |  |  |  |  |  |  |  |  |
| Lack of organizational infrastructure |  |  |  |  |  |  |  |  |  |  |  |
| Lack of financial support |  |  |  |  |  |  |  |  |  |  |  |
| Lack of local OPAT protocol |  |  |  |  |  |  |  |  |  |  |  |
| Absence of enough experience/expertise available |  |  |  |  |  |  |  |  |  |  |  |
| Lack OPAT training |  |  |  |  |  |  |  |  |  |  |  |
| Lack of administrative support |  |  |  |  |  |  |  |  |  |  |  |
| Lack of communication and coordination among OPAT providers |  |  |  |  |  |  |  |  |  |  |  |
| Lack of a system for laboratory monitoring and patient follow-up |  |  |  |  |  |  |  |  |  |  |  |
| Lack of team-based OPAT program |  |  |  |  |  |  |  |  |  |  |  |
| Lack of ownership over patient cases |  |  |  |  |  |  |  |  |  |  |  |
| Poor availability of antimicrobials and administration devices |  |  |  |  |  |  |  |  |  |  |  |
| Poor stability of antimicrobials |  |  |  |  |  |  |  |  |  |  |  |
| Dosing frequency of antimicrobials |  |  |  |  |  |  |  |  |  |  |  |
| Problem in screening (selection) of candidate patient |  |  |  |  |  |  |  |  |  |  |  |
| Diverse geographic locations of patients |  |  |  |  |  |  |  |  |  |  |  |
| Inadequate patient environment |  |  |  |  |  |  |  |  |  |  |  |
| Prescription difficulties |  |  |  |  |  |  |  |  |  |  |  |
| Lack of or delay in reimbursement |  |  |  |  |  |  |  |  |  |  |  |

1. **Facilitators of the OPAT program**

What advantages does OPAT offer compared to inpatient parenteral antimicrobial treatment?

Please rate your level of agreement on a scale from 0 (completely disagree) to 10 (completely agree).

|  | 0 | 1 | 2 | 3 | 4 | 5 | 6 | 7 | 8 | 9 | 10 |
| --- | --- | --- | --- | --- | --- | --- | --- | --- | --- | --- | --- |
| Reduction in length of hospital stay |  |  |  |  |  |  |  |  |  |  |  |
| Free hospital beds |  |  |  |  |  |  |  |  |  |  |  |
| Enhance patient quality of life |  |  |  |  |  |  |  |  |  |  |  |
| Cost-effectiveness |  |  |  |  |  |  |  |  |  |  |  |
| Comparable clinical effectiveness |  |  |  |  |  |  |  |  |  |  |  |
| Comparable safety |  |  |  |  |  |  |  |  |  |  |  |
| Patient preference/ satisfaction |  |  |  |  |  |  |  |  |  |  |  |

We plan to conduct a follow-up qualitative study to further explore the barriers and facilitators of the OPAT program in detail. Would you be interested in receiving further information about this future study to see if you would be interested in participating?

Yes

No

The survey team expresses gratitude for your time and effort in responding to our questionnaire. Your answers will be taken into consideration to contribute to the improvement of the OPAT program. Additionally, you will receive an update once the survey results become available.

Thank you!

Supplementary Table 1: Barriers to OPAT service implementation

| Barriers | Completely disagree | | Strongly disagree | | Very much disagree | | Disagree | | Somewhat disagree | | Neither agree nor disagree | | Somewhat agree | | Agree | | Strongly agree | | Very strongly agree | | Completely agree | |
| --- | --- | --- | --- | --- | --- | --- | --- | --- | --- | --- | --- | --- | --- | --- | --- | --- | --- | --- | --- | --- | --- | --- |
|  | N | % | N | % | N | % | N | % | N | % | N | % | N | % | N | % | N | % | N | % | N | % |
| Lack of legal framework | 60 | 43.5% | 17 | 12.3% | 13 | 9.4% | 3 | 2.2% | 5 | 3.6% | 13 | 9.4% | 7 | 5.1% | 10 | 7.2% | 4 | 2.9% | 3 | 2.2% | 3 | 2.2% |
| Lack of organizational infrastructure | 41 | 29.7% | 12 | 8.7% | 7 | 5.1% | 9 | 6.5% | 6 | 4.3% | 17 | 12.3% | 9 | 6.5% | 8 | 5.8% | 13 | 9.4% | 5 | 3.6% | 11 | 8.0% |
| Lack of financial support | 38 | 27.5% | 6 | 4.3% | 5 | 3.6% | 8 | 5.8% | 4 | 2.9% | 18 | 13.0% | 9 | 6.5% | 8 | 5.8% | 17 | 12.3% | 7 | 5.1% | 18 | 13.0% |
| Lack of local OPAT protocol | 52 | 37.7% | 14 | 10.1% | 15 | 10.9% | 5 | 3.6% | 9 | 6.5% | 15 | 10.9% | 8 | 5.8% | 5 | 3.6% | 8 | 5.8% | 4 | 2.9% | 3 | 2.2% |
| Absence of enough experience/expertise available | 50 | 36.2% | 17 | 12.3% | 14 | 10.1% | 10 | 7.2% | 2 | 1.4% | 15 | 10.9% | 6 | 4.3% | 8 | 5.8% | 5 | 3.6% | 7 | 5.1% | 4 | 2.9% |
| Lack of OPAT training | 47 | 34.1% | 17 | 12.3% | 13 | 9.4% | 7 | 5.1% | 7 | 5.1% | 17 | 12.3% | 9 | 6.5% | 7 | 5.1% | 7 | 5.1% | 4 | 2.9% | 3 | 2.2% |
| Lack of administrative support | 45 | 32.6% | 14 | 10.1% | 9 | 6.5% | 8 | 5.8% | 5 | 3.6% | 16 | 11.6% | 12 | 8.7% | 5 | 3.6% | 7 | 5.1% | 11 | 8.0% | 6 | 4.3% |
| Lack of communication and coordination among OPAT providers | 42 | 30.4% | 16 | 11.6% | 15 | 10.9% | 9 | 6.5% | 7 | 5.1% | 17 | 12.3% | 10 | 7.2% | 7 | 5.1% | 3 | 2.2% | 7 | 5.1% | 5 | 3.6% |
| Lack of a system for laboratory monitoring and patient follow-up | 62 | 44.9% | 21 | 15.2% | 10 | 7.2% | 8 | 5.8% | 3 | 2.2% | 15 | 10.9% | 6 | 4.3% | 4 | 2.9% | 4 | 2.9% | 4 | 2.9% | 1 | 0.7% |
| Lack of team-based OPAT program | 56 | 40.6% | 18 | 13.0% | 8 | 5.8% | 4 | 2.9% | 7 | 5.1% | 14 | 10.1% | 7 | 5.1% | 6 | 4.3% | 9 | 6.5% | 6 | 4.3% | 3 | 2.2% |
| Lack of ownership over patient cases | 48 | 35.0% | 14 | 10.2% | 2 | 1.5% | 10 | 7.3% | 10 | 7.3% | 19 | 13.9% | 13 | 9.5% | 4 | 2.9% | 7 | 5.1% | 6 | 4.4% | 4 | 2.9% |
| Poor availability of antimicrobials and administration devices | 43 | 31.2% | 22 | 15.9% | 13 | 9.4% | 4 | 2.9% | 5 | 3.6% | 15 | 10.9% | 14 | 10.1% | 4 | 2.9% | 3 | 2.2% | 6 | 4.3% | 9 | 6.5% |
| Poor stability of antimicrobials | 34 | 24.6% | 14 | 10.1% | 12 | 8.7% | 15 | 10.9% | 10 | 7.2% | 12 | 8.7% | 16 | 11.6% | 7 | 5.1% | 7 | 5.1% | 6 | 4.3% | 5 | 3.6% |
| Dosing frequency of antimicrobials | 23 | 16.7% | 13 | 9.4% | 10 | 7.2% | 14 | 10.1% | 7 | 5.1% | 15 | 10.9% | 20 | 14.5% | 9 | 6.5% | 9 | 6.5% | 11 | 8.0% | 7 | 5.1% |
| Problem in the screening (selection) of candidate patients | 40 | 29.0% | 16 | 11.6% | 8 | 5.8% | 10 | 7.2% | 10 | 7.2% | 21 | 15.2% | 10 | 7.2% | 9 | 6.5% | 6 | 4.3% | 3 | 2.2% | 5 | 3.6% |
| Diverse geographic locations of patients | 24 | 17.4% | 9 | 6.5% | 6 | 4.3% | 6 | 4.3% | 10 | 7.2% | 13 | 9.4% | 15 | 10.9% | 19 | 13.8% | 17 | 12.3% | 9 | 6.5% | 10 | 7.2% |
| Inadequate patient environment | 24 | 17.4% | 17 | 12.3% | 16 | 11.6% | 10 | 7.2% | 6 | 4.3% | 15 | 10.9% | 22 | 15.9% | 8 | 5.8% | 8 | 5.8% | 6 | 4.3% | 6 | 4.3% |
| Prescription difficulties | 54 | 39.1% | 27 | 19.6% | 15 | 10.9% | 6 | 4.3% | 5 | 3.6% | 16 | 11.6% | 6 | 4.3% | 3 | 2.2% | 2 | 1.4% | 2 | 1.4% | 2 | 1.4% |
| Lack of or delay in reimbursement | 75 | 54.3% | 15 | 10.9% | 6 | 4.3% | 7 | 5.1% | 13 | 9.4% | 12 | 8.7% | 3 | 2.2% | 2 | 1.4% | 2 | 1.4% | 2 | 1.4% | 1 | 0.7% |

Supplementary Table 2: Facilitators to OPAT service implementation

| Facilitators | Completely disagree | | Strongly disagree | | Very much disagree | | Disagree | | Somewhat disagree | | Neither agree nor disagree | | Somewhat agree | | Agree | | Strongly agree | | Very strongly agree | | Completely agree | |
| --- | --- | --- | --- | --- | --- | --- | --- | --- | --- | --- | --- | --- | --- | --- | --- | --- | --- | --- | --- | --- | --- | --- |
|  | N | % | N | % | N | % | N | % | N | % | N | % | N | % | N | % | N | % | N | % | N | % |
| Reduction in length of hospital stay | 0 | 0.0% | 1 | 0.7% | 0 | 0.0% | 1 | 0.7% | 0 | 0.0% | 2 | 1.5% | 3 | 2.2% | 0 | 0.0% | 6 | 4.5% | 19 | 14.2% | 102 | 76.1% |
| Free hospital beds | 1 | 0.8% | 0 | 0.0% | 1 | 0.8% | 0 | 0.0% | 3 | 2.3% | 3 | 2.3% | 3 | 2.3% | 2 | 1.5% | 8 | 6.0% | 16 | 12.0% | 96 | 72.2% |
| Enhance patient quality of life | 0 | 0.0% | 0 | 0.0% | 0 | 0.0% | 0 | 0.0% | 0 | 0.0% | 2 | 1.5% | 6 | 4.5% | 1 | 0.8% | 11 | 8.3% | 20 | 15.0% | 93 | 69.9% |
| Cost-effectiveness | 0 | 0.0% | 0 | 0.0% | 0 | 0.0% | 2 | 1.5% | 2 | 1.5% | 6 | 4.5% | 7 | 5.3% | 12 | 9.0% | 19 | 14.3% | 14 | 10.5% | 71 | 53.4% |
| Comparable clinical effectiveness | 0 | 0.0% | 0 | 0.0% | 0 | 0.0% | 1 | 0.8% | 3 | 2.3% | 2 | 1.5% | 6 | 4.5% | 5 | 3.8% | 14 | 10.5% | 21 | 15.8% | 81 | 60.9% |
| Comparable safety | 0 | 0.0% | 0 | 0.0% | 1 | 0.8% | 0 | 0.0% | 4 | 3.0% | 4 | 3.0% | 9 | 6.8% | 9 | 6.8% | 19 | 14.3% | 27 | 20.3% | 60 | 45.1% |
| Patient preference/ satisfaction | 0 | 0.0% | 0 | 0.0% | 0 | 0.0% | 0 | 0.0% | 0 | 0.0% | 3 | 2.3% | 4 | 3.0% | 8 | 6.0% | 11 | 8.3% | 31 | 23.3% | 76 | 57.1% |

Supplementary Table 3: Characteristics of OPAT services (Spain)

| Characteristics | | Frequency | Percent |
| --- | --- | --- | --- |
| Location of OPAT centre (n=52) | | | |
|  | Urban | 39 | 75 |
|  | Suburban | 8 | 15.4 |
|  | Rural | 5 | 9.6 |
| Type of healthcare facility (n=52) | | | |
|  | University teaching hospital | 44 | 84.6 |
|  | Community teaching hospital | 5 | 9.6 |
|  | Nonteaching hospital | 3 | 5.8 |
| Scope of OPAT program (n=52) | | | |
|  | Adult OPAT | 39 | 75 |
|  | Paediatric OPAT | 2 | 3.8 |
|  | Adult and paediatric OPAT | 11 | 21.2 |
| Number of patients managed by the OPAT program monthly (n=52) | | | |
|  | 1-5 patients per month | 1 | 1.9 |
|  | 6-15 patients per month | 5 | 9.6 |
|  | 16-25 patients per month | 11 | 21.2 |
|  | 26-50 patients per month | 11 | 21.2 |
|  | More than 50 patients per month | 24 | 46.2 |
| Formal OPAT service structure (n=52) | | | |
|  | Yes | 40 | 76.9 |
|  | No | 12 | 23.1 |
| Members of the OPAT team | | | |
|  | Acute physician | 32 | 80 |
|  | Clinical pharmacist | 23 | 57.5 |
|  | Specialist nurse | 19 | 47.5 |
|  | Infectious diseases physician | 18 | 45 |
|  | Administrative assistant | 15 | 37.5 |
|  | Advanced nurse practitioner | 12 | 30 |
|  | Community nurse | 9 | 22.5 |
|  | Clinical microbiologist | 7 | 17.5 |
|  | Consultant paediatrician | 3 | 7.5 |
|  | IT specialist | 2 | 5 |
|  | Other staff* | 8 | 20 |
| Clinical leader of OPAT team (n=40) | | | |
|  | No formal lead | 6 | 15 |
|  | Acute physician | 21 | 52.7 |
|  | Infectious disease physician | 9 | 22.5 |
|  | Clinical microbiologist | 1 | 2.5 |
|  | Other** | 3 | 7.5 |
| OPAT program falls under the responsibility of the antimicrobial stewardship team (n=51) | | | |
|  | Yes | 21 | 41.5 |
|  | No | 30 | 58.8 |
| Type of OPAT model of care (n=51) | | | |
|  | H-OPAT | 25 | 49 |
|  | S-OPAT | 4 | 7.8 |
|  | H-OPAT and S-OPAT | 16 | 31.4 |
|  | H-OPAT and C-OPAT | 2 | 3.9 |
|  | All models | 4 | 7.8 |
| Mandatory ID physician consultation before discharging a patient (n=51) | | | |
|  | Yes | 39 | 76.5 |
|  | No | 12 | 23.5 |
| Patients have been discharged with OPAT without seeing any member of the ID team (n=51) | | | |
|  | Yes | 8 | 15.7 |
|  | No | 43 | 84.3 |
| Monitoring patients for antimicrobial toxicity (n=51) | | | |
|  | Yes | 45 | 86.5 |
|  | No | 6 | 11.5 |
| Frequency of patients' antimicrobial toxicity monitoring (n=45) | | | |
|  | Rarely | 4 | 8.9 |
|  | Once a week | 15 | 33.3 |
|  | Twice a week | 4 | 8.9 |
|  | It depends on the antimicrobial | 22 | 48.9 |
| Systematic method of tracking patient monitoring (n=45) | | | |
|  | Yes | 37 | 82.2 |
|  | No | 8 | 17.8 |
| Systematic method of ensuring patient follow-up (n=51) | | | |
|  | Yes | 44 | 86.3 |
|  | No | 7 | 13.7 |
| Training program for patients and caregivers who choose to self-administer OPAT (n=51) | | | |
|  | Yes | 24 | 47.1 |
|  | No | 27 | 52.9 |
| Audit of OPAT program (n=51) | | | |
|  | Yes | 7 | 13.7 |
|  | No | 44 | 86.3 |
| Outcome measure to measure the OPAT program | | | |
|  | Emergency department visit during OPAT | 6 | 85.7 |
|  | Completion of OPAT as planned | 5 | 71.4 |
|  | Side effects of antimicrobials | 5 | 71.4 |
|  | Clinical cure | 5 | 71.4 |
|  | Readmission rate | 5 | 71.4 |
|  | Complication | 5 | 71.4 |
|  | Patient satisfaction | 4 | 57.1 |
|  | Patient survival | 4 | 57.1 |
|  | Laboratory monitoring | 4 | 57.1 |

Other*Pharmacy technician, Hospital at Home physicians, Pneumology specialist, Geriatric specialist, family physician, social worker; other**internist

Supplementary Table 4: Barriers to OPAT services implementation (Spain)

| Barriers | Completely disagree | | Strongly disagree | | Very much disagree | | disagree | | Somewhat disagree | | Neither agree nor disagree | | Somewhat agree | | Agree | | Strongly agree | | Very strongly agree | | Completely agree | |
| --- | --- | --- | --- | --- | --- | --- | --- | --- | --- | --- | --- | --- | --- | --- | --- | --- | --- | --- | --- | --- | --- | --- |
|  | N | % | N | % | N | % | N | % | N | % | N | % | N | % | N | % | N | % | N | % | N | % |
| Lack of legal framework (n=49) | 22 | 44.9% | 6 | 12.2% | 3 | 6.1% | 1 | 2.0% | 1 | 2.0% | 6 | 12.2% | 2 | 4.1% | 3 | 6.1% | 2 | 4.1% | 2 | 4.1% | 1 | 2.0% |
| Lack of organizational infrastructure (n=49) | 16 | 32.7% | 3 | 6.1% | 2 | 4.1% | 6 | 12.2% | 1 | 2.0% | 6 | 12.2% | 1 | 2.0% | 3 | 6.1% | 7 | 14.3% | 2 | 4.1% | 2 | 4.1% |
| Lack of financial support (n=49) | 18 | 36.7% | 5 | 10.2% | 1 | 2.0% | 1 | 2.0% | 0 | 0.0% | 6 | 12.2% | 2 | 4.1% | 2 | 4.1% | 6 | 12.2% | 4 | 8.2% | 4 | 8.2% |
| Lack of local OPAT protocol (n=49) | 17 | 34.7% | 8 | 16.3% | 5 | 10.2% | 1 | 2.0% | 1 | 2.0% | 5 | 10.2% | 4 | 8.2% | 3 | 6.1% | 2 | 4.1% | 2 | 4.1% | 1 | 2.0% |
| Absence of enough experience/expertise available (n=49) | 22 | 44.9% | 6 | 12.2% | 6 | 12.2% | 3 | 6.1% | 0 | 0.0% | 3 | 6.1% | 2 | 4.1% | 3 | 6.1% | 1 | 2.0% | 2 | 4.1% | 1 | 2.0% |
| Lack of OPAT training (n=49) | 17 | 34.7% | 6 | 12.2% | 6 | 12.2% | 2 | 4.1% | 3 | 6.1% | 7 | 14.3% | 3 | 6.1% | 3 | 6.1% | 1 | 2.0% | 1 | 2.0% | 0 | 0.0% |
| Lack of administrative support (n=49) | 18 | 36.7% | 6 | 12.2% | 4 | 8.2% | 0 | 0.0% | 2 | 4.1% | 8 | 16.3% | 3 | 6.1% | 3 | 6.1% | 1 | 2.0% | 2 | 4.1% | 2 | 4.1% |
| Lack of communication and coordination among OPAT providers (n=49) | 15 | 30.6% | 5 | 10.2% | 4 | 8.2% | 2 | 4.1% | 4 | 8.2% | 8 | 16.3% | 1 | 2.0% | 4 | 8.2% | 1 | 2.0% | 3 | 6.1% | 2 | 4.1% |
| Lack of a system for laboratory monitoring and patient follow-up (n=49) | 22 | 44.9% | 8 | 16.3% | 4 | 8.2% | 5 | 10.2% | 0 | 0.0% | 5 | 10.2% | 2 | 4.1% | 2 | 4.1% | 1 | 2.0% | 0 | 0.0% | 0 | 0.0% |
| Lack of team-based OPAT program (n=49) | 18 | 36.7% | 8 | 16.3% | 2 | 4.1% | 1 | 2.0% | 3 | 6.1% | 4 | 8.2% | 4 | 8.2% | 3 | 6.1% | 6 | 12.2% | 0 | 0.0% | 0 | 0.0% |
| Lack of ownership over patient cases (n=48) | 20 | 41.7% | 6 | 12.5% | 0 | 0.0% | 4 | 8.3% | 4 | 8.3% | 3 | 6.3% | 4 | 8.3% | 3 | 6.3% | 4 | 8.3% | 0 | 0.0% | 0 | 0.0% |
| Poor availability of antimicrobials and administration devices (n=49) | 19 | 38.8% | 12 | 24.5% | 4 | 8.2% | 3 | 6.1% | 1 | 2.0% | 6 | 12.2% | 2 | 4.1% | 1 | 2.0% | 1 | 2.0% | 0 | 0.0% | 0 | 0.0% |
| Poor stability of antimicrobials | 12 | 24.5% | 8 | 16.3% | 5 | 10.2% | 5 | 10.2% | 2 | 4.1% | 3 | 6.1% | 5 | 10.2% | 3 | 6.1% | 3 | 6.1% | 3 | 6.1% | 0 | 0.0% |
| Dosing frequency of antimicrobials (n=49) | 12 | 24.5% | 9 | 18.4% | 4 | 8.2% | 3 | 6.1% | 1 | 2.0% | 5 | 10.2% | 6 | 12.2% | 3 | 6.1% | 2 | 4.1% | 4 | 8.2% | 0 | 0.0% |
| Problem in the screening (selection) of candidate patients (n=49) | 13 | 26.5% | 6 | 12.2% | 3 | 6.1% | 5 | 10.2% | 5 | 10.2% | 8 | 16.3% | 3 | 6.1% | 3 | 6.1% | 3 | 6.1% | 0 | 0.0% | 0 | 0.0% |
| Diverse geographic locations of patients (n=49) | 12 | 24.5% | 5 | 10.2% | 3 | 6.1% | 2 | 4.1% | 2 | 4.1% | 1 | 2.0% | 4 | 8.2% | 7 | 14.3% | 7 | 14.3% | 3 | 6.1% | 3 | 6.1% |
| Inadequate patient environment (n=49) | 3 | 6.1% | 5 | 10.2% | 9 | 18.4% | 5 | 10.2% | 3 | 6.1% | 4 | 8.2% | 9 | 18.4% | 5 | 10.2% | 3 | 6.1% | 2 | 4.1% | 1 | 2.0% |
| Prescription difficulties | 19 | 38.8% | 14 | 28.6% | 5 | 10.2% | 1 | 2.0% | 3 | 6.1% | 6 | 12.2% | 1 | 2.0% | 0 | 0.0% | 0 | 0.0% | 0 | 0.0% | 0 | 0.0% |
| Lack of or delay in reimbursement (n=49) | 31 | 63.3% | 5 | 10.2% | 2 | 4.1% | 2 | 4.1% | 5 | 10.2% | 3 | 6.1% | 0 | 0.0% | 1 | 2.0% | 0 | 0.0% | 0 | 0.0% | 0 | 0.0% |

Supplementary Table 5: Facilitators of OPAT services implementation (Spain)

| Facilitators | Completely disagree | | Strongly disagree | | Very much disagree | | disagree | | Somewhat disagree | | Neither agree nor disagree | | Somewhat agree | | Agree | | Strongly agree | | Very strongly agree | | Completely agree | |
| --- | --- | --- | --- | --- | --- | --- | --- | --- | --- | --- | --- | --- | --- | --- | --- | --- | --- | --- | --- | --- | --- | --- |
|  | N | % | N | % | N | % | N | % | N | % | N | % | N | % | N | % | N | % | N | % | N | % |
| Reduction in length of hospital stay (n=48) | 0 | 0.0% | 1 | 2.1% | 0 | 0.0% | 1 | 2.1% | 0 | 0.0% | 0 | 0.0% | 1 | 2.1% | 0 | 0.0% | 1 | 2.1% | 8 | 16.7% | 36 | 75.0% |
| Free hospital beds (n=48) | 1 | 2.1% | 0 | 0.0% | 1 | 2.1% | 0 | 0.0% | 2 | 4.2% | 3 | 6.3% | 1 | 2.1% | 1 | 2.1% | 2 | 4.2% | 6 | 12.5% | 31 | 64.6% |
| Enhance patient quality of life (n=48) | 0 | 0.0% | 0 | 0.0% | 0 | 0.0% | 0 | 0.0% | 0 | 0.0% | 0 | 0.0% | 0 | 0.0% | 0 | 0.0% | 4 | 8.3% | 8 | 16.7% | 36 | 75.0% |
| Cost-effectiveness (n=48) | 0 | 0.0% | 0 | 0.0% | 0 | 0.0% | 0 | 0.0% | 0 | 0.0% | 1 | 2.1% | 1 | 2.1% | 5 | 10.4% | 8 | 16.7% | 5 | 10.4% | 28 | 58.3% |
| Comparable clinical effectiveness (n=48) | 0 | 0.0% | 0 | 0.0% | 0 | 0.0% | 0 | 0.0% | 0 | 0.0% | 0 | 0.0% | 0 | 0.0% | 2 | 4.2% | 2 | 4.2% | 7 | 14.6% | 37 | 77.1% |
| Comparable safety (n=48) | 0 | 0.0% | 0 | 0.0% | 0 | 0.0% | 0 | 0.0% | 2 | 4.2% | 0 | 0.0% | 0 | 0.0% | 4 | 8.3% | 6 | 12.5% | 10 | 20.8% | 26 | 54.2% |
| Patient preference/ satisfaction (n=48) | 0 | 0.0% | 0 | 0.0% | 0 | 0.0% | 0 | 0.0% | 0 | 0.0% | 0 | 0.0% | 0 | 0.0% | 1 | 2.1% | 2 | 4.2% | 15 | 31.3% | 30 | 62.5% |

Supplementary Table 6: Characteristics of OPAT services (United Kingdom)

| Characteristics | | Frequency | Percent |
| --- | --- | --- | --- |
| Location of OPAT centre (n=43) | | | |
|  | Urban | 25 | 58.1 |
|  | Suburban | 9 | 20.9 |
|  | Rural | 9 | 20.9 |
| Type of healthcare facility (n=44) | | | |
|  | University teaching hospital | 23 | 52.3 |
|  | Community teaching hospital | 8 | 18.2 |
|  | Nonteaching hospital | 6 | 13.6 |
|  | Other^£^ | 7 | 15.9 |
| Scope of OPAT program (n=44) | | | |
|  | Adult OPAT | 40 | 90.9 |
|  | Adult and paediatric OPAT | 4 | 9.1 |
| Number of patients managed by the OPAT program monthly (n=44) | | | |
|  | 1-5 patients per month | 5 | 11.4 |
|  | 6-15 patients per month | 9 | 20.5 |
|  | 16-25 patients per month | 11 | 25 |
|  | 26-50 patients per month | 14 | 31.8 |
|  | More than 50 patients per month | 8 | 11.4 |
| Formal OPAT service structure (n=53) | | | |
|  | Yes | 35 | 81.4 |
|  | No | 8 | 18.6 |
| Members of the OPAT team | | | |
|  | Infectious diseases physician | 27 | 77.1 |
|  | Clinical pharmacist | 25 | 71.4 |
|  | Specialist nurse | 27 | 77.1 |
|  | Clinical microbiologist | 23 | 65.7 |
|  | Advanced nurse practitioner | 10 | 28.6 |
|  | Administrative assistant | 14 | 40 |
|  | Community nurse | 13 | 37.1 |
|  | Acute physician | 9 | 25.7 |
|  | Other staff* | 8 | 22.9 |
| Clinical leader of OPAT team (n=35) | | | |
|  | No formal lead | 2 | 5.7 |
|  | Infectious disease physician | 23 | 65.7 |
|  | Clinical microbiologist | 5 | 14.3 |
|  | Nurse | 4 | 11.4 |
|  | Clinical pharmacist | 1 | 2.8 |
| OPAT program falls under the responsibility of the antimicrobial stewardship team (n=43) | | | |
|  | Yes | 30 | 69.8 |
|  | No | 13 | 30.2 |
| Type of OPAT model of care (n=43) | | | |
|  | H-OPAT | 4 | 9.3 |
|  | C-OPAT | 3 | 7 |
|  | S-OPAT | 1 | 2.3 |
|  | H-OPAT and S-OPAT | 6 | 14 |
|  | S-OPAT and C-OPAT | 4 | 9.3 |
|  | H-OPAT and C-OPAT | 3 | 7 |
|  | All models | 22 | 51.2 |
| Mandatory ID physician consultation before discharging a patient (n=43) | | | |
|  | Yes | 22 | 51.2 |
|  | No | 21 | 48.8 |
| Patients have been discharged with OPAT without seeing any member of the ID team (n=43) | | | |
|  | Yes | 25 | 58.1 |
|  | No | 18 | 41.9 |
| Monitoring patients for antimicrobial toxicity (n=43) | | | |
|  | Yes | 39 | 90.7 |
|  | No | 4 | 9.3 |
| Frequency of patients' antimicrobial toxicity monitoring (n=39) | | | |
|  | Rarely | 21 | 53.8 |
|  | Once a week | 1 | 2.6 |
|  | Twice a week | 17 | 43.6 |
|  | It depends on the antimicrobial |  |  |
| Systematic method of tracking patient monitoring (n=39) | | | |
|  | Yes | 31 | 79.5 |
|  | No | 8 | 20.5 |
| Systematic method of ensuring patient follow-up (n=43) | | | |
|  | Yes | 37 | 86 |
|  | No | 6 | 14 |
| Training program for patients and caregivers who choose to self-administer OPAT (n=43) | | | |
|  | Yes | 33 | 76.7 |
|  | No | 10 | 23.3 |
| Audit of OPAT program (n=43) | | | |
|  | Yes | 33 | 76.7 |
|  | No | 10 | 23.3 |
| Outcome measure to measure the OPAT program | | | |
|  | Complication | 27 | 81.8 |
|  | Completion of OPAT as planned | 30 | 90.9 |
|  | Side effects of antimicrobials | 24 | 72.7 |
|  | Clinical cure | 24 | 72.7 |
|  | Patient satisfaction | 22 | 66.7 |
|  | Readmission rate | 21 | 63.6 |
|  | Emergency department visit during OPAT | 13 | 39.4 |
|  | Laboratory monitoring | 16 | 48.5 |
|  | Patient survival | 13 | 39.4 |
|  | Other* | 3 | 9.1 |

^£^Community team, general hospital, community admission avoidance rapid response, cure hospital, Other*pharmacist, vascular access team, pharmacy technician; other*Patient Demographics, Rate of bacteraemias, History of MDR infection and microbiological isolates/organisms being treated, staffing, elastomeric pump ordering

Supplementary Table 7: Barriers to OPAT services implementation (United Kingdom, n=38)

| Barriers | Completely disagree | | Strongly disagree | | Very much disagree | | disagree | | Somewhat disagree | | Neither agree nor disagree | | Somewhat agree | | Agree | | Strongly agree | | Very strongly agree | | Completely agree | |
| --- | --- | --- | --- | --- | --- | --- | --- | --- | --- | --- | --- | --- | --- | --- | --- | --- | --- | --- | --- | --- | --- | --- |
|  | N | % | N | % | N | % | N | % | N | % | N | % | N | % | N | % | N | % | N | % | N | % |
| Lack of legal framework | 23 | 60.5% | 5 | 13.2% | 3 | 7.9% | 1 | 2.6% | 0 | 0.0% | 3 | 7.9% | 1 | 2.6% | 1 | 2.6% | 0 | 0.0% | 0 | 0.0% | 1 | 2.6% |
| Lack of organizational infrastructure | 14 | 36.8% | 3 | 7.9% | 0 | 0.0% | 2 | 5.3% | 2 | 5.3% | 5 | 13.2% | 4 | 10.5% | 3 | 7.9% | 0 | 0.0% | 2 | 5.3% | 3 | 7.9% |
| Lack of financial support | 8 | 21.1% | 0 | 0.0% | 2 | 5.3% | 3 | 7.9% | 1 | 2.6% | 6 | 15.8% | 4 | 10.5% | 1 | 2.6% | 3 | 7.9% | 2 | 5.3% | 8 | 21.1% |
| Lack of local OPAT protocol | 21 | 55.3% | 2 | 5.3% | 3 | 7.9% | 0 | 0.0% | 2 | 5.3% | 6 | 15.8% | 1 | 2.6% | 0 | 0.0% | 2 | 5.3% | 1 | 2.6% | 0 | 0.0% |
| Absence of enough experience/expertise available | 16 | 42.1% | 5 | 13.2% | 1 | 2.6% | 3 | 7.9% | 1 | 2.6% | 5 | 13.2% | 1 | 2.6% | 3 | 7.9% | 0 | 0.0% | 2 | 5.3% | 1 | 2.6% |
| Lack of OPAT training | 18 | 47.4% | 4 | 10.5% | 2 | 5.3% | 2 | 5.3% | 0 | 0.0% | 6 | 15.8% | 1 | 2.6% | 4 | 10.5% | 1 | 2.6% | 0 | 0.0% | 0 | 0.0% |
| Lack of administrative support | 14 | 36.8% | 2 | 5.3% | 1 | 2.6% | 4 | 10.5% | 3 | 7.9% | 3 | 7.9% | 2 | 5.3% | 1 | 2.6% | 3 | 7.9% | 3 | 7.9% | 2 | 5.3% |
| Lack of communication and coordination among OPAT providers | 15 | 39.5% | 2 | 5.3% | 5 | 13.2% | 3 | 7.9% | 2 | 5.3% | 4 | 10.5% | 5 | 13.2% | 0 | 0.0% | 2 | 5.3% | 0 | 0.0% | 0 | 0.0% |
| Lack of a system for laboratory monitoring and patient follow-up | 23 | 60.5% | 3 | 7.9% | 1 | 2.6% | 2 | 5.3% | 1 | 2.6% | 5 | 13.2% | 1 | 2.6% | 1 | 2.6% | 1 | 2.6% | 0 | 0.0% | 0 | 0.0% |
| Lack of team-based OPAT program | 23 | 60.5% | 5 | 13.2% | 2 | 5.3% | 1 | 2.6% | 1 | 2.6% | 2 | 5.3% | 0 | 0.0% | 0 | 0.0% | 1 | 2.6% | 2 | 5.3% | 1 | 2.6% |
| Lack of ownership over patient cases | 15 | 39.5% | 1 | 2.6% | 1 | 2.6% | 3 | 7.9% | 3 | 7.9% | 8 | 21.1% | 2 | 5.3% | 1 | 2.6% | 2 | 5.3% | 0 | 0.0% | 2 | 5.3% |
| Poor availability of antimicrobials and administration devices | 12 | 31.6% | 3 | 7.9% | 4 | 10.5% | 0 | 0.0% | 2 | 5.3% | 3 | 7.9% | 9 | 23.7% | 0 | 0.0% | 0 | 0.0% | 1 | 2.6% | 4 | 10.5% |
| Poor stability of antimicrobials | 13 | 34.2% | 3 | 7.9% | 4 | 10.5% | 5 | 13.2% | 1 | 2.6% | 3 | 7.9% | 5 | 13.2% | 0 | 0.0% | 1 | 2.6% | 1 | 2.6% | 2 | 5.3% |
| Dosing frequency of antimicrobials | 6 | 15.8% | 3 | 7.9% | 3 | 7.9% | 6 | 15.8% | 2 | 5.3% | 3 | 7.9% | 8 | 21.1% | 1 | 2.6% | 3 | 7.9% | 2 | 5.3% | 1 | 2.6% |
| Problem in the screening (selection) of candidate patients | 19 | 50.0% | 3 | 7.9% | 3 | 7.9% | 2 | 5.3% | 2 | 5.3% | 5 | 13.2% | 2 | 5.3% | 2 | 5.3% | 0 | 0.0% | 0 | 0.0% | 0 | 0.0% |
| Diverse geographic locations of patients | 9 | 23.7% | 2 | 5.3% | 2 | 5.3% | 2 | 5.3% | 3 | 7.9% | 6 | 15.8% | 3 | 7.9% | 5 | 13.2% | 4 | 10.5% | 1 | 2.6% | 1 | 2.6% |
| Inadequate patient environment | 14 | 36.8% | 5 | 13.2% | 4 | 10.5% | 2 | 5.3% | 2 | 5.3% | 5 | 13.2% | 4 | 10.5% | 1 | 2.6% | 0 | 0.0% | 0 | 0.0% | 1 | 2.6% |
| Prescription difficulties | 20 | 52.6% | 6 | 15.8% | 5 | 13.2% | 2 | 5.3% | 1 | 2.6% | 2 | 5.3% | 1 | 2.6% | 1 | 2.6% | 0 | 0.0% | 0 | 0.0% | 0 | 0.0% |
| Lack of or delay in reimbursement | 24 | 63.2% | 4 | 10.5% | 0 | 0.0% | 4 | 10.5% | 2 | 5.3% | 2 | 5.3% | 1 | 2.6% | 0 | 0.0% | 0 | 0.0% | 1 | 2.6% | 0 | 0.0% |

Supplementary Table 8: Facilitators of OPAT services implementation (United Kingdom)

| Facilitators | Completely disagree | | Strongly disagree | | Very much disagree | | Disagree | | Somewhat disagree | | Neither agree nor disagree | | Somewhat agree | | Agree | | Strongly agree | | Very strongly agree | | Completely agree | |
| --- | --- | --- | --- | --- | --- | --- | --- | --- | --- | --- | --- | --- | --- | --- | --- | --- | --- | --- | --- | --- | --- | --- |
|  | N | % | N | % | N | % | N | % | N | % | N | % | N | % | N | % | N | % | N | % | N | % |
| Reduction in length of hospital stay (n=36) | 0 | 0.0% | 0 | 0.0% | 0 | 0.0% | 0 | 0.0% | 0 | 0.0% | 1 | 2.8% | 1 | 2.8% | 0 | 0.0% | 2 | 5.6% | 1 | 2.8% | 31 | 86.1% |
| Free hospital beds (n=35) | 0 | 0.0% | 0 | 0.0% | 0 | 0.0% | 0 | 0.0% | 0 | 0.0% | 0 | 0.0% | 1 | 2.9% | 0 | 0.0% | 2 | 5.7% | 1 | 2.9% | 31 | 88.6% |
| Enhance patient quality of life (n=35) | 0 | 0.0% | 0 | 0.0% | 0 | 0.0% | 0 | 0.0% | 0 | 0.0% | 0 | 0.0% | 2 | 5.7% | 0 | 0.0% | 4 | 11.4% | 1 | 2.9% | 28 | 80.0% |
| Cost-effectiveness (n=35) | 0 | 0.0% | 0 | 0.0% | 0 | 0.0% | 1 | 2.9% | 1 | 2.9% | 0 | 0.0% | 3 | 8.6% | 4 | 11.4% | 6 | 17.1% | 0 | 0.0% | 20 | 57.1% |
| Comparable clinical effectiveness (n=35) | 0 | 0.0% | 0 | 0.0% | 0 | 0.0% | 0 | 0.0% | 1 | 2.9% | 0 | 0.0% | 3 | 8.6% | 2 | 5.7% | 5 | 14.3% | 1 | 2.9% | 23 | 65.7% |
| Comparable safety (n=35) | 0 | 0.0% | 0 | 0.0% | 0 | 0.0% | 0 | 0.0% | 0 | 0.0% | 0 | 0.0% | 3 | 8.6% | 3 | 8.6% | 8 | 22.9% | 3 | 8.6% | 18 | 51.4% |
| Patient preference/ satisfaction (n=35) | 0 | 0.0% | 0 | 0.0% | 0 | 0.0% | 0 | 0.0% | 0 | 0.0% | 0 | 0.0% | 1 | 2.9% | 1 | 2.9% | 4 | 11.4% | 5 | 14.3% | 24 | 68.6% |

Supplementary Table 9: Characteristics of OPAT services (Australia)

| Characteristics | | Frequency | Percent |
| --- | --- | --- | --- |
| Location of OPAT centre (n=18) | | | |
|  | Urban | 11 | 61.1 |
|  | Suburban | 4 | 22.2 |
|  | Rural | 3 | 16.7 |
| Type of healthcare facility (n=18) | | | |
|  | University teaching hospital | 14 | 77.8 |
|  | Community teaching hospital | 2 | 11.1 |
|  | Nonteaching hospital | 1 | 5.6 |
|  | Other^£^ | 1 | 5.6 |
| Scope of OPAT program (n=18) | | | |
|  | Adult OPAT | 8 | 44.4 |
|  | Paediatric OPAT | 4 | 22.2 |
|  | Adult and paediatric OPAT | 6 | 33.3 |
| Number of patients managed by the OPAT program monthly (n=18) | | | |
|  | 6-15 patients per month | 2 | 11.1 |
|  | 16-25 patients per month | 3 | 16.7 |
|  | 26-50 patients per month | 7 | 38.9 |
|  | More than 50 patients per month | 8 | 33.3 |
| Formal OPAT service structure (n=18) | | | |
|  | Yes | 17 | 94.4 |
|  | No | 1 | 5.6 |
| Members of the OPAT team | | | |
|  | Infectious diseases physician | 11 | 64.7 |
|  | Specialist nurse | 11 | 64.7 |
|  | Clinical pharmacist | 11 | 64.7 |
|  | Administrative assistant | 11 | 64.7 |
|  | Acute physician | 10 | 58.8 |
|  | Community nurse | 9 | 52.9 |
|  | Consultant paediatrician | 6 | 35.3 |
|  | Advanced nurse practitioner | 3 | 17.6 |
|  | Clinical microbiologist | 1 | 5.9 |
|  | IT specialist | 1 | 5.9 |
|  | Other staff* | 3 | 17.6 |
| Clinical leader of OPAT team (n=17) | | | |
|  | No formal lead | 6 | 35.3 |
|  | Infectious disease physician | 4 | 23.5 |
|  | Clinical microbiologist | 3 | 17.6 |
|  | Acute physician | 3 | 17.6 |
|  | Other** | 1 | 5.9 |
| OPAT program falls under the responsibility of the antimicrobial stewardship team (n=18) | | | |
|  | Yes | 9 | 50 |
|  | No | 9 | 50 |
| Type of OPAT model of care (n=18) | | | |
|  | H-OPAT | 7 | 38.9 |
|  | H-OPAT and C-OPAT | 6 | 33.3 |
|  | H-OPAT and S-OPAT | 2 | 11.1 |
|  | S-OPAT and C-OPAT | 1 | 5.6 |
|  | All models | 2 | 11.1 |
| Mandatory ID physician consultation before discharging a patient (n=18) | | | |
|  | Yes | 11 | 61.1 |
|  | No | 7 | 38.9 |
| Patients have been discharged with OPAT without seeing any member of the ID team (n=18) | | | |
|  | Yes | 12 | 66.7 |
|  | No | 6 | 33.3 |
| Monitoring patients for antimicrobial toxicity (n=18) | | | |
|  | Yes | 16 | 88.9 |
|  | No | 2 | 11.1 |
| Frequency of patients' antimicrobial toxicity monitoring (n=16) | | | |
|  | Rarely |  |  |
|  | Once a week | 6 | 37.5 |
|  | Twice a week | 1 | 6.3 |
|  | It depends on the antimicrobial | 9 | 56.3 |
| Systematic method of tracking patient monitoring (n=16) | | | |
|  | Yes | 14 | 77.8 |
|  | No | 2 | 11.1 |
| Systematic method of ensuring patient follow-up (n=18) | | | |
|  | Yes | 13 | 72.2 |
|  | No | 5 | 27.8 |
| Training program for patients and caregivers who choose to self-administer OPAT (n=18) | | | |
|  | Yes | 6 | 33.3 |
|  | No | 12 | 66.6 |
| Audit of OPAT program (n=51) | | | |
|  | Yes | 8 | 44.4 |
|  | No | 10 | 55.6 |
| Outcome measure to measure the OPAT program | | | |
|  | Completion of OPAT as planned | 6 | 75 |
|  | Readmission rate | 5 | 62.5 |
|  | Side effects of antimicrobials | 2 | 25 |
|  | Patient satisfaction | 3 | 37.5 |
|  | Emergency department visit during OPAT | 2 | 25 |
|  | Complication | 2 | 25 |
|  | Laboratory monitoring | 2 | 25 |
|  | Clinical cure | 1 | 12.5 |
|  | Other*** | 1 | 12.5 |

^£^Private independent provider, Other*general practitioners, care coordinators, allied health clinicians, skilled nurse coordinators and nurse clinicians, physiotherapist; other**physicians/GPs; other***adherence to evidence-based guidelines

Supplementary Table 10: Barriers to OPAT services implementation (Australia, n=16)

| Barriers | Completely disagree | | Strongly disagree | | Very much disagree | | Disagree | | Somewhat disagree | | Neither agree nor disagree | | Somewhat agree | | Agree | | Strongly agree | | Very strongly agree | | Completely agree | |
| --- | --- | --- | --- | --- | --- | --- | --- | --- | --- | --- | --- | --- | --- | --- | --- | --- | --- | --- | --- | --- | --- | --- |
|  | N | % | N | % | N | % | N | % | N | % | N | % | N | % | N | % | N | % | N | % | N | % |
| Lack of legal framework | 8 | 50.0% | 3 | 18.8% | 3 | 18.8% | 0 | 0.0% | 0 | 0.0% | 0 | 0.0% | 1 | 6.3% | 1 | 6.3% | 0 | 0.0% | 0 | 0.0% | 0 | 0.0% |
| Lack of organizational infrastructure | 6 | 37.5% | 2 | 12.5% | 1 | 6.3% | 0 | 0.0% | 3 | 18.8% | 0 | 0.0% | 1 | 6.3% | 1 | 6.3% | 1 | 6.3% | 0 | 0.0% | 1 | 6.3% |
| Lack of financial support | 7 | 43.8% | 0 | 0.0% | 2 | 12.5% | 1 | 6.3% | 2 | 12.5% | 0 | 0.0% | 1 | 6.3% | 1 | 6.3% | 2 | 12.5% | 0 | 0.0% | 0 | 0.0% |
| Lack of local OPAT protocol | 7 | 43.8% | 2 | 12.5% | 3 | 18.8% | 0 | 0.0% | 2 | 12.5% | 0 | 0.0% | 1 | 6.3% | 0 | 0.0% | 0 | 0.0% | 1 | 6.3% | 0 | 0.0% |
| Absence of enough experience/expertise available | 8 | 50.0% | 4 | 25.0% | 2 | 12.5% | 0 | 0.0% | 0 | 0.0% | 1 | 6.3% | 0 | 0.0% | 0 | 0.0% | 0 | 0.0% | 1 | 6.3% | 0 | 0.0% |
| Lack of OPAT training | 7 | 43.8% | 4 | 25.0% | 2 | 12.5% | 0 | 0.0% | 1 | 6.3% | 2 | 12.5% | 0 | 0.0% | 0 | 0.0% | 0 | 0.0% | 0 | 0.0% | 0 | 0.0% |
| Lack of administrative support | 8 | 50.0% | 3 | 18.8% | 2 | 12.5% | 1 | 6.3% | 0 | 0.0% | 0 | 0.0% | 2 | 12.5% | 0 | 0.0% | 0 | 0.0% | 0 | 0.0% | 0 | 0.0% |
| Lack of communication and coordination among OPAT providers | 7 | 43.8% | 5 | 31.3% | 0 | 0.0% | 1 | 6.3% | 0 | 0.0% | 0 | 0.0% | 2 | 12.5% | 1 | 6.3% | 0 | 0.0% | 0 | 0.0% | 0 | 0.0% |
| Lack of a system for laboratory monitoring and patient follow-up | 8 | 50.0% | 3 | 18.8% | 2 | 12.5% | 0 | 0.0% | 1 | 6.3% | 0 | 0.0% | 1 | 6.3% | 0 | 0.0% | 0 | 0.0% | 0 | 0.0% | 1 | 6.3% |
| Lack of team-based OPAT program | 9 | 56.3% | 1 | 6.3% | 2 | 12.5% | 1 | 6.3% | 3 | 18.8% | 0 | 0.0% | 0 | 0.0% | 0 | 0.0% | 0 | 0.0% | 0 | 0.0% | 0 | 0.0% |
| Lack of ownership over patient cases | 8 | 50.0% | 3 | 18.8% | 1 | 6.3% | 0 | 0.0% | 1 | 6.3% | 1 | 6.3% | 0 | 0.0% | 0 | 0.0% | 1 | 6.3% | 1 | 6.3% | 0 | 0.0% |
| Poor availability of antimicrobials and administration devices | 9 | 56.3% | 4 | 25.0% | 2 | 12.5% | 0 | 0.0% | 1 | 6.3% | 0 | 0.0% | 0 | 0.0% | 0 | 0.0% | 0 | 0.0% | 0 | 0.0% | 0 | 0.0% |
| Poor stability of antimicrobials | 5 | 31.3% | 1 | 6.3% | 2 | 12.5% | 1 | 6.3% | 2 | 12.5% | 0 | 0.0% | 4 | 25.0% | 1 | 6.3% | 0 | 0.0% | 0 | 0.0% | 0 | 0.0% |
| Dosing frequency of antimicrobials | 3 | 18.8% | 1 | 6.3% | 2 | 12.5% | 3 | 18.8% | 3 | 18.8% | 0 | 0.0% | 3 | 18.8% | 0 | 0.0% | 0 | 0.0% | 0 | 0.0% | 1 | 6.3% |
| Problem in the screening (selection) of candidate patients | 4 | 25.0% | 4 | 25.0% | 2 | 12.5% | 0 | 0.0% | 1 | 6.3% | 2 | 12.5% | 1 | 6.3% | 0 | 0.0% | 0 | 0.0% | 0 | 0.0% | 2 | 12.5% |
| Diverse geographic locations of patients | 2 | 12.5% | 1 | 6.3% | 1 | 6.3% | 1 | 6.3% | 2 | 12.5% | 1 | 6.3% | 3 | 18.8% | 2 | 12.5% | 1 | 6.3% | 1 | 6.3% | 1 | 6.3% |
| Inadequate patient environment | 3 | 18.8% | 2 | 12.5% | 2 | 12.5% | 1 | 6.3% | 1 | 6.3% | 1 | 6.3% | 5 | 31.3% | 0 | 0.0% | 0 | 0.0% | 0 | 0.0% | 1 | 6.3% |
| Prescription difficulties | 9 | 56.3% | 4 | 25.0% | 1 | 6.3% | 0 | 0.0% | 0 | 0.0% | 2 | 12.5% | 0 | 0.0% | 0 | 0.0% | 0 | 0.0% | 0 | 0.0% | 0 | 0.0% |
| Lack of or delay in reimbursement | 12 | 75.0% | 2 | 12.5% | 0 | 0.0% | 0 | 0.0% | 1 | 6.3% | 1 | 6.3% | 0 | 0.0% | 0 | 0.0% | 0 | 0.0% | 0 | 0.0% | 0 | 0.0% |

Supplementary Table 11: Facilitators of OPAT services implementation (Australia, n=16)

| Facilitators | Completely disagree | | Strongly disagree | | Very much disagree | | disagree | | Somewhat disagree | | Neither agree nor disagree | | Somewhat agree | | Agree | | Strongly agree | | Very strongly agree | | Completely agree | |
| --- | --- | --- | --- | --- | --- | --- | --- | --- | --- | --- | --- | --- | --- | --- | --- | --- | --- | --- | --- | --- | --- | --- |
|  | N | % | N | % | N | % | N | % | N | % | N | % | N | % | N | % | N | % | N | % | N | % |
| Reduction in length of hospital stay | 0 | 0.0% | 0 | 0.0% | 0 | 0.0% | 0 | 0.0% | 0 | 0.0% | 0 | 0.0% | 0 | 0.0% | 0 | 0.0% | 2 | 12.5% | 2 | 12.5% | 12 | 75.0% |
| Free hospital beds | 0 | 0.0% | 0 | 0.0% | 0 | 0.0% | 0 | 0.0% | 0 | 0.0% | 0 | 0.0% | 0 | 0.0% | 0 | 0.0% | 2 | 12.5% | 2 | 12.5% | 12 | 75.0% |
| Enhance patient quality of life | 0 | 0.0% | 0 | 0.0% | 0 | 0.0% | 0 | 0.0% | 0 | 0.0% | 0 | 0.0% | 1 | 6.3% | 0 | 0.0% | 1 | 6.3% | 3 | 18.8% | 11 | 68.8% |
| Cost-effectiveness | 0 | 0.0% | 0 | 0.0% | 0 | 0.0% | 0 | 0.0% | 0 | 0.0% | 1 | 6.3% | 0 | 0.0% | 1 | 6.3% | 2 | 12.5% | 3 | 18.8% | 9 | 56.3% |
| Comparable clinical effectiveness | 0 | 0.0% | 0 | 0.0% | 0 | 0.0% | 0 | 0.0% | 0 | 0.0% | 0 | 0.0% | 1 | 6.3% | 0 | 0.0% | 2 | 12.5% | 6 | 37.5% | 7 | 43.8% |
| Comparable safety | 0 | 0.0% | 0 | 0.0% | 0 | 0.0% | 0 | 0.0% | 0 | 0.0% | 0 | 0.0% | 3 | 18.8% | 0 | 0.0% | 3 | 18.8% | 5 | 31.3% | 5 | 31.3% |
| Patient preference/ satisfaction | 0 | 0.0% | 0 | 0.0% | 0 | 0.0% | 0 | 0.0% | 0 | 0.0% | 0 | 0.0% | 1 | 6.3% | 0 | 0.0% | 2 | 12.5% | 4 | 25.0% | 9 | 56.3% |

Supplementary Table 12: Characteristics of OPAT services (Turkey)

| Characteristics | | Frequency | Percent |
| --- | --- | --- | --- |
| Location of OPAT centre (n=12) | | | |
|  | Urban | 12 | 100 |
| Type of healthcare facility (n=13) | | | |
|  | University teaching hospital | 12 | 92.3 |
|  | Community teaching hospital | 1 | 7.7 |
| Scope of OPAT program (n=13) | | | |
|  | Adult OPAT | 9 | 69.2 |
|  | Adult and paediatric OPAT | 4 | 30.8 |
| Number of patients managed by the OPAT program monthly (n=13) | | | |
|  | 1-5 patients per month | 6 | 46.2 |
|  | 6-15 patients per month | 2 | 15.4 |
|  | 16-25 patients per month | 3 | 23.1 |
|  | More than 50 patients per month | 2 | 15.4 |
| Formal OPAT service structure (n=12) | | | |
|  | Yes | 5 | 41.7 |
|  | No | 7 | 58.3 |
| Members of the OPAT team | | | |
|  | Infectious diseases physician | 5 | 100 |
|  | Specialist nurse | 4 | 80 |
|  | Clinical microbiologist | 1 | 20 |
|  | Advanced nurse practitioner | 1 | 20 |
|  | Clinical pharmacist | 1 | 20 |
|  | Administrative assistant | 1 | 20 |
| Clinical leader of OPAT team (n=5) | | | |
|  | Infectious disease physician | 5 | 100 |
| OPAT program falls under the responsibility of the antimicrobial stewardship team (n=12) | | | |
|  | Yes | 9 | 75 |
|  | No | 3 | 25 |
| Type of OPAT model of care (n=12) | | | |
|  | C-OPAT | 7 | 58.3 |
|  | S-OPAT | 1 | 8.3 |
|  | H-OPAT and C-OPAT | 1 | 8.3 |
|  | H-OPAT and S-OPAT | 1 | 8.3 |
|  | S-OPAT and S-OPAT | 1 | 8.3 |
|  | All models | 1 | 8.3 |
| Mandatory ID physician consultation before discharging a patient (n=12) | | | |
|  | Yes | 11 | 91.7 |
|  | No | 1 | 8.3 |
| Patients have been discharged with OPAT without seeing any member of the ID team (n=12) | | | |
|  | Yes | 3 | 25 |
|  | No | 9 | 75 |
| Monitoring patients for antimicrobial toxicity (n=12) | | | |
|  | Yes | 8 | 66.7 |
|  | No | 4 | 33.3 |
| Frequency of patients' antimicrobial toxicity monitoring (n=8) | | | |
|  | Once a week | 2 | 25 |
|  | Twice a week | 2 | 25 |
|  | It depends on the antimicrobial | 4 | 50 |
| Systematic method of tracking patient monitoring (n=8) | | | |
|  | Yes | 7 | 87.5 |
|  | No | 1 | 12.5 |
| Systematic method of ensuring patient follow-up (n=12) | | | |
|  | Yes | 9 | 75 |
|  | No | 3 | 25 |
| Training program for patients and caregivers who choose to self-administer OPAT (n=12) | | | |
|  | Yes | 2 | 16.7 |
|  | No | 10 | 83.3 |
| Audit of OPAT program (n=12) | | | |
|  | Yes | 2 | 16.7 |
|  | No | 10 | 83.3 |
| Outcome measure to measure the OPAT program | | | |
|  | Completion of OPAT as planned | 2 |  |
|  | Side effects of antimicrobials | 2 |  |
|  | Clinical cure | 2 |  |
|  | Readmission rate | 1 |  |
|  | Complication | 1 |  |
|  | Laboratory monitoring | 1 |  |

Supplementary Table 13: Barriers to OPAT services implementation (Turkey, n=12)

| Barriers | Completely disagree | | Strongly disagree | | Very much disagree | | Disagree | | Somewhat disagree | | Neither agree nor disagree | | Somewhat agree | | Agree | | Strongly agree | | Very strongly agree | | Completely agree | |
| --- | --- | --- | --- | --- | --- | --- | --- | --- | --- | --- | --- | --- | --- | --- | --- | --- | --- | --- | --- | --- | --- | --- |
|  | N | % | N | % | N | % | N | % | N | % | N | % | N | % | N | % | N | % | N | % | N | % |
| Lack of legal framework | 2 | 16.7% | 0 | 0.0% | 0 | 0.0% | 1 | 8.3% | 2 | 16.7% | 2 | 16.7% | 1 | 8.3% | 1 | 8.3% | 2 | 16.7% | 1 | 8.3% | 0 | 0.0% |
| Lack of organizational infrastructure | 0 | 0.0% | 1 | 8.3% | 0 | 0.0% | 1 | 8.3% | 0 | 0.0% | 1 | 8.3% | 1 | 8.3% | 1 | 8.3% | 4 | 33.3% | 0 | 0.0% | 3 | 25.0% |
| Lack of financial support | 0 | 0.0% | 1 | 8.3% | 0 | 0.0% | 1 | 8.3% | 1 | 8.3% | 3 | 25.0% | 1 | 8.3% | 2 | 16.7% | 3 | 25.0% | 0 | 0.0% | 0 | 0.0% |
| Lack of local OPAT protocol | 1 | 8.3% | 1 | 8.3% | 0 | 0.0% | 1 | 8.3% | 1 | 8.3% | 2 | 16.7% | 0 | 0.0% | 1 | 8.3% | 4 | 33.3% | 0 | 0.0% | 1 | 8.3% |
| Absence of enough experience/expertise available | 1 | 8.3% | 0 | 0.0% | 2 | 16.7% | 3 | 25.0% | 0 | 0.0% | 2 | 16.7% | 1 | 8.3% | 0 | 0.0% | 1 | 8.3% | 2 | 16.7% | 0 | 0.0% |
| Lack OPAT training | 1 | 8.3% | 0 | 0.0% | 0 | 0.0% | 2 | 16.7% | 2 | 16.7% | 0 | 0.0% | 1 | 8.3% | 0 | 0.0% | 2 | 16.7% | 3 | 25.0% | 1 | 8.3% |
| Lack of administrative support | 0 | 0.0% | 1 | 8.3% | 0 | 0.0% | 1 | 8.3% | 0 | 0.0% | 2 | 16.7% | 2 | 16.7% | 0 | 0.0% | 2 | 16.7% | 3 | 25.0% | 1 | 8.3% |
| Lack of communication and coordination among OPAT providers | 0 | 0.0% | 0 | 0.0% | 3 | 25.0% | 1 | 8.3% | 1 | 8.3% | 2 | 16.7% | 0 | 0.0% | 1 | 8.3% | 0 | 0.0% | 3 | 25.0% | 1 | 8.3% |
| Lack of a system for laboratory monitoring and patient follow-up | 1 | 8.3% | 2 | 16.7% | 0 | 0.0% | 0 | 0.0% | 1 | 8.3% | 3 | 25.0% | 1 | 8.3% | 0 | 0.0% | 1 | 8.3% | 3 | 25.0% | 0 | 0.0% |
| Lack of team-based OPAT program | 2 | 16.7% | 1 | 8.3% | 1 | 8.3% | 0 | 0.0% | 0 | 0.0% | 2 | 16.7% | 0 | 0.0% | 2 | 16.7% | 1 | 8.3% | 2 | 16.7% | 1 | 8.3% |
| Lack of ownership over patient cases | 0 | 0.0% | 2 | 16.7% | 0 | 0.0% | 0 | 0.0% | 0 | 0.0% | 3 | 25.0% | 4 | 33.3% | 0 | 0.0% | 0 | 0.0% | 3 | 25.0% | 0 | 0.0% |
| Poor availability of antimicrobials and administration devices | 1 | 8.3% | 1 | 8.3% | 1 | 8.3% | 0 | 0.0% | 0 | 0.0% | 1 | 8.3% | 2 | 16.7% | 2 | 16.7% | 1 | 8.3% | 3 | 25.0% | 0 | 0.0% |
| Poor stability of antimicrobials | 1 | 8.3% | 1 | 8.3% | 0 | 0.0% | 3 | 25.0% | 2 | 16.7% | 2 | 16.7% | 0 | 0.0% | 1 | 8.3% | 1 | 8.3% | 1 | 8.3% | 0 | 0.0% |
| Dosing frequency of antimicrobials | 0 | 0.0% | 0 | 0.0% | 0 | 0.0% | 1 | 8.3% | 0 | 0.0% | 2 | 16.7% | 3 | 25.0% | 0 | 0.0% | 3 | 25.0% | 3 | 25.0% | 0 | 0.0% |
| Problem in screening (selection) of candidate patients | 0 | 0.0% | 1 | 8.3% | 0 | 0.0% | 2 | 16.7% | 1 | 8.3% | 3 | 25.0% | 4 | 33.3% | 1 | 8.3% | 0 | 0.0% | 0 | 0.0% | 0 | 0.0% |
| Diverse geographic locations of patients | 0 | 0.0% | 1 | 8.3% | 0 | 0.0% | 1 | 8.3% | 1 | 8.3% | 2 | 16.7% | 1 | 8.3% | 1 | 8.3% | 3 | 25.0% | 2 | 16.7% | 0 | 0.0% |
| Inadequate patient environment | 1 | 8.3% | 2 | 16.7% | 0 | 0.0% | 2 | 16.7% | 0 | 0.0% | 0 | 0.0% | 2 | 16.7% | 0 | 0.0% | 2 | 16.7% | 3 | 25.0% | 0 | 0.0% |
| Prescription difficulties | 1 | 8.3% | 0 | 0.0% | 2 | 16.7% | 0 | 0.0% | 1 | 8.3% | 1 | 8.3% | 2 | 16.7% | 1 | 8.3% | 2 | 16.7% | 2 | 16.7% | 0 | 0.0% |
| Lack of or delay in reimbursement | 1 | 8.3% | 1 | 8.3% | 1 | 8.3% | 1 | 8.3% | 1 | 8.3% | 3 | 25.0% | 1 | 8.3% | 1 | 8.3% | 1 | 8.3% | 1 | 8.3% | 0 | 0.0% |

Supplementary Table 14: Facilitators of OPAT services implementation (Turkey)

| Facilitators | Completely disagree | | Strongly disagree | | Very much disagree | | Disagree | | Somewhat disagree | | Neither agree nor disagree | | Somewhat agree | | Agree | | Strongly agree | | Very strongly agree | | Completely agree | |
| --- | --- | --- | --- | --- | --- | --- | --- | --- | --- | --- | --- | --- | --- | --- | --- | --- | --- | --- | --- | --- | --- | --- |
|  | N | % | N | % | N | % | N | % | N | % | N | % | N | % | N | % | N | % | N | % | N | % |
| Reduction in the length of hospital stay | 0 | 0.0% | 0 | 0.0% | 0 | 0.0% | 0 | 0.0% | 0 | 0.0% | 1 | 8.3% | 1 | 8.3% | 0 | 0.0% | 1 | 8.3% | 4 | 33.3% | 5 | 41.7% |
| Free hospital beds | 0 | 0.0% | 0 | 0.0% | 0 | 0.0% | 0 | 0.0% | 1 | 8.3% | 0 | 0.0% | 1 | 8.3% | 1 | 8.3% | 2 | 16.7% | 2 | 16.7% | 5 | 41.7% |
| Enhance patient quality of life | 0 | 0.0% | 0 | 0.0% | 0 | 0.0% | 0 | 0.0% | 0 | 0.0% | 1 | 8.3% | 3 | 25.0% | 1 | 8.3% | 1 | 8.3% | 2 | 16.7% | 4 | 33.3% |
| Cost-effectiveness | 0 | 0.0% | 0 | 0.0% | 0 | 0.0% | 1 | 8.3% | 1 | 8.3% | 0 | 0.0% | 1 | 8.3% | 1 | 8.3% | 1 | 8.3% | 2 | 16.7% | 5 | 41.7% |
| Comparable clinical effectiveness | 0 | 0.0% | 0 | 0.0% | 0 | 0.0% | 1 | 8.3% | 2 | 16.7% | 1 | 8.3% | 2 | 16.7% | 0 | 0.0% | 1 | 8.3% | 1 | 8.3% | 4 | 33.3% |
| Comparable safety | 0 | 0.0% | 0 | 0.0% | 1 | 8.3% | 0 | 0.0% | 2 | 16.7% | 2 | 16.7% | 2 | 16.7% | 0 | 0.0% | 1 | 8.3% | 2 | 16.7% | 2 | 16.7% |
| Patient preference/ satisfaction | 0 | 0.0% | 0 | 0.0% | 0 | 0.0% | 0 | 0.0% | 0 | 0.0% | 2 | 16.7% | 1 | 8.3% | 3 | 25.0% | 3 | 25.0% | 0 | 0.0% | 3 | 25.0% |

Supplementary Table 15: Characteristics of OPAT services (Malaysia)

| Characteristics | | Frequency | Percent |
| --- | --- | --- | --- |
| Location of OPAT centre (n=16) | | | |
|  | Urban | 13 | 81.3 |
|  | Suburban | 3 | 18.8 |
|  | Rural |  |  |
| Type of healthcare facility (n=16) | | | |
|  | University teaching hospital | 1 | 6.3 |
|  | Community teaching hospital | 5 | 31.3 |
|  | Nonteaching hospital | 10 | 62.5 |
| Scope of OPAT program (n=16) | | | |
|  | Adult OPAT | 15 | 93.8 |
|  | Adult and paediatric OPAT | 1 | 6.3 |
| Number of patients managed by the OPAT program monthly (n=16) | | | |
|  | 1-5 patients per month | 16 | 100 |
| Formal OPAT service structure (n=16) | | | |
|  | Yes | 15 | 93.8 |
|  | No | 1 | 6.3 |
| Members of the OPAT team | | | |
|  | Infectious diseases physician | 15 | 100 |
|  | Clinical pharmacist | 13 | 86.7 |
|  | Acute physician | 4 | 26.7 |
|  | Specialist nurse | 4 | 26.7 |
|  | Community nurse | 4 | 26.7 |
|  | Clinical microbiologist | 3 | 20 |
|  | Consultant paediatrician | 1 | 6.7 |
|  | Advanced nurse practitioner | 1 | 6.7 |
|  | Administrative assistant | 1 | 6.7 |
|  | Other staff* | 7 | 46.7 |
| Clinical leader of OPAT team (n=15) | | | |
|  | Infectious disease physician | 14 | 93.3 |
|  | Other** | 1 | 6.7 |
| OPAT program falls under the responsibility of the antimicrobial stewardship team (n=16) | | | |
|  | Yes | 13 | 81.3 |
|  | No | 3 | 18.8 |
| Type of OPAT model of care (n=16) | | | |
|  | C-OPAT | 16 | 100 |
| Mandatory ID physician consultation before discharging a patient (n=16) | | | |
|  | Yes | 15 | 93.8 |
|  | No | 1 | 6.3 |
| Patients have been discharged with OPAT without seeing any member of the ID team (n=16) | | | |
|  | Yes | 1 | 6.3 |
|  | No | 15 | 93.8 |
| Monitoring patients for antimicrobial toxicity (n=16) | | | |
|  | Yes | 12 | 75 |
|  | No | 4 | 25 |
| Frequency of patients' antimicrobial toxicity monitoring (n=12) | | | |
|  | Once a week | 4 | 33.3 |
|  | It depends on the antimicrobial | 8 | 66.7 |
| Systematic method of tracking patient monitoring (n=12) | | | |
|  | Yes | 9 | 75 |
|  | No | 3 | 25 |
| Systematic method of ensuring patient follow-up (n=16) | | | |
|  | Yes | 13 | 81.3 |
|  | No | 3 | 18.8 |
| Training program for patients and caregivers who choose to self-administer OPAT (n=16) | | | |
|  | Yes | 13 | 81.3 |
|  | No | 3 | 18.8 |
| Audit of OPAT program (n=16) | | | |
|  | Yes | 3 | 18.8 |
|  | No | 13 | 81.3 |
| Outcome measure to measure the OPAT program | | | |
|  | Completion of OPAT as planned | 3 |  |
|  | Readmission rate | 2 |  |
|  | Complication | 2 |  |
|  | Patient satisfaction | 2 |  |
|  | Side effects of antimicrobials | 1 |  |
|  | Clinical cure | 1 |  |
|  | Emergency department visit during OPAT | 1 |  |
|  | Patient survival | 1 |  |
|  | Laboratory monitoring | 1 |  |
|  | Other*** | 1 |  |

Other*medical assistant, medical specialist; other**medical specialist; other***Reasons of decline OPAT service

Supplementary Table 16: Barriers to OPAT services implementation (Malaysia, n=16)

| Barriers | Completely disagree | | Strongly disagree | | Very much disagree | | Disagree | | Somewhat disagree | | Neither agree nor disagree | | Somewhat agree | | Agree | | Strongly agree | | Very strongly agree | | Completely agree | |
| --- | --- | --- | --- | --- | --- | --- | --- | --- | --- | --- | --- | --- | --- | --- | --- | --- | --- | --- | --- | --- | --- | --- |
|  | N | % | N | % | N | % | N | % | N | % | N | % | N | % | N | % | N | % | N | % | N | % |
| Lack of legal framework | 2 | 16.7% | 0 | 0.0% | 0 | 0.0% | 1 | 8.3% | 2 | 16.7% | 2 | 16.7% | 1 | 8.3% | 1 | 8.3% | 2 | 16.7% | 1 | 8.3% | 0 | 0.0% |
| Lack of organizational infrastructure | 0 | 0.0% | 1 | 8.3% | 0 | 0.0% | 1 | 8.3% | 0 | 0.0% | 1 | 8.3% | 1 | 8.3% | 1 | 8.3% | 4 | 33.3% | 0 | 0.0% | 3 | 25.0% |
| Lack of financial support | 0 | 0.0% | 1 | 8.3% | 0 | 0.0% | 1 | 8.3% | 1 | 8.3% | 3 | 25.0% | 1 | 8.3% | 2 | 16.7% | 3 | 25.0% | 0 | 0.0% | 0 | 0.0% |
| Lack of local OPAT protocol | 1 | 8.3% | 1 | 8.3% | 0 | 0.0% | 1 | 8.3% | 1 | 8.3% | 2 | 16.7% | 0 | 0.0% | 1 | 8.3% | 4 | 33.3% | 0 | 0.0% | 1 | 8.3% |
| Absence of enough experience/expertise available | 1 | 8.3% | 0 | 0.0% | 2 | 16.7% | 3 | 25.0% | 0 | 0.0% | 2 | 16.7% | 1 | 8.3% | 0 | 0.0% | 1 | 8.3% | 2 | 16.7% | 0 | 0.0% |
| Lack of OPAT training | 1 | 8.3% | 0 | 0.0% | 0 | 0.0% | 2 | 16.7% | 2 | 16.7% | 0 | 0.0% | 1 | 8.3% | 0 | 0.0% | 2 | 16.7% | 3 | 25.0% | 1 | 8.3% |
| Lack of administrative support | 0 | 0.0% | 1 | 8.3% | 0 | 0.0% | 1 | 8.3% | 0 | 0.0% | 2 | 16.7% | 2 | 16.7% | 0 | 0.0% | 2 | 16.7% | 3 | 25.0% | 1 | 8.3% |
| Lack of communication and coordination among OPAT providers | 0 | 0.0% | 0 | 0.0% | 3 | 25.0% | 1 | 8.3% | 1 | 8.3% | 2 | 16.7% | 0 | 0.0% | 1 | 8.3% | 0 | 0.0% | 3 | 25.0% | 1 | 8.3% |
| Lack of a system for laboratory monitoring and patient follow-up | 1 | 8.3% | 2 | 16.7% | 0 | 0.0% | 0 | 0.0% | 1 | 8.3% | 3 | 25.0% | 1 | 8.3% | 0 | 0.0% | 1 | 8.3% | 3 | 25.0% | 0 | 0.0% |
| Lack of team-based OPAT program | 2 | 16.7% | 1 | 8.3% | 1 | 8.3% | 0 | 0.0% | 0 | 0.0% | 2 | 16.7% | 0 | 0.0% | 2 | 16.7% | 1 | 8.3% | 2 | 16.7% | 1 | 8.3% |
| Lack of ownership over patient cases | 0 | 0.0% | 2 | 16.7% | 0 | 0.0% | 0 | 0.0% | 0 | 0.0% | 3 | 25.0% | 4 | 33.3% | 0 | 0.0% | 0 | 0.0% | 3 | 25.0% | 0 | 0.0% |
| Poor availability of antimicrobials and administration devices | 1 | 8.3% | 1 | 8.3% | 1 | 8.3% | 0 | 0.0% | 0 | 0.0% | 1 | 8.3% | 2 | 16.7% | 2 | 16.7% | 1 | 8.3% | 3 | 25.0% | 0 | 0.0% |
| Poor stability of antimicrobials | 1 | 8.3% | 1 | 8.3% | 0 | 0.0% | 3 | 25.0% | 2 | 16.7% | 2 | 16.7% | 0 | 0.0% | 1 | 8.3% | 1 | 8.3% | 1 | 8.3% | 0 | 0.0% |
| Dosing frequency of antimicrobials | 0 | 0.0% | 0 | 0.0% | 0 | 0.0% | 1 | 8.3% | 0 | 0.0% | 2 | 16.7% | 3 | 25.0% | 0 | 0.0% | 3 | 25.0% | 3 | 25.0% | 0 | 0.0% |
| Problem in the screening (selection) of candidate patients | 0 | 0.0% | 1 | 8.3% | 0 | 0.0% | 2 | 16.7% | 1 | 8.3% | 3 | 25.0% | 4 | 33.3% | 1 | 8.3% | 0 | 0.0% | 0 | 0.0% | 0 | 0.0% |
| Diverse geographic locations of patients | 0 | 0.0% | 1 | 8.3% | 0 | 0.0% | 1 | 8.3% | 1 | 8.3% | 2 | 16.7% | 1 | 8.3% | 1 | 8.3% | 3 | 25.0% | 2 | 16.7% | 0 | 0.0% |
| Inadequate patient environment | 1 | 8.3% | 2 | 16.7% | 0 | 0.0% | 2 | 16.7% | 0 | 0.0% | 0 | 0.0% | 2 | 16.7% | 0 | 0.0% | 2 | 16.7% | 3 | 25.0% | 0 | 0.0% |
| Prescription difficulties | 1 | 8.3% | 0 | 0.0% | 2 | 16.7% | 0 | 0.0% | 1 | 8.3% | 1 | 8.3% | 2 | 16.7% | 1 | 8.3% | 2 | 16.7% | 2 | 16.7% | 0 | 0.0% |
| Lack of or delay in reimbursement | 1 | 8.3% | 1 | 8.3% | 1 | 8.3% | 1 | 8.3% | 1 | 8.3% | 3 | 25.0% | 1 | 8.3% | 1 | 8.3% | 1 | 8.3% | 1 | 8.3% | 0 | 0.0% |

Supplementary Table 17: Facilitators of OPAT services implementation (Malaysia=15)

| Facilitators | Completely disagree | | Strongly disagree | | Very much disagree | | Disagree | | Somewhat disagree | | Neither agree nor disagree | | Somewhat agree | | Agree | | Strongly agree | | Very strongly agree | | Completely agree | |
| --- | --- | --- | --- | --- | --- | --- | --- | --- | --- | --- | --- | --- | --- | --- | --- | --- | --- | --- | --- | --- | --- | --- |
|  | N | % | N | % | N | % | N | % | N | % | N | % | N | % | N | % | N | % | N | % | N | % |
| Reduction in length of hospital stay | 0 | 0.0% | 0 | 0.0% | 0 | 0.0% | 0 | 0.0% | 0 | 0.0% | 1 | 8.3% | 1 | 8.3% | 0 | 0.0% | 1 | 8.3% | 4 | 33.3% | 5 | 41.7% |
| Free hospital beds | 0 | 0.0% | 0 | 0.0% | 0 | 0.0% | 0 | 0.0% | 1 | 8.3% | 0 | 0.0% | 1 | 8.3% | 1 | 8.3% | 2 | 16.7% | 2 | 16.7% | 5 | 41.7% |
| Enhance patient quality of life | 0 | 0.0% | 0 | 0.0% | 0 | 0.0% | 0 | 0.0% | 0 | 0.0% | 1 | 8.3% | 3 | 25.0% | 1 | 8.3% | 1 | 8.3% | 2 | 16.7% | 4 | 33.3% |
| Cost-effectiveness | 0 | 0.0% | 0 | 0.0% | 0 | 0.0% | 1 | 8.3% | 1 | 8.3% | 0 | 0.0% | 1 | 8.3% | 1 | 8.3% | 1 | 8.3% | 2 | 16.7% | 5 | 41.7% |
| Comparable clinical effectiveness | 0 | 0.0% | 0 | 0.0% | 0 | 0.0% | 1 | 8.3% | 2 | 16.7% | 1 | 8.3% | 2 | 16.7% | 0 | 0.0% | 1 | 8.3% | 1 | 8.3% | 4 | 33.3% |
| Comparable safety | 0 | 0.0% | 0 | 0.0% | 1 | 8.3% | 0 | 0.0% | 2 | 16.7% | 2 | 16.7% | 2 | 16.7% | 0 | 0.0% | 1 | 8.3% | 2 | 16.7% | 2 | 16.7% |
| Patient preference/ satisfaction | 0 | 0.0% | 0 | 0.0% | 0 | 0.0% | 0 | 0.0% | 0 | 0.0% | 2 | 16.7% | 1 | 8.3% | 3 | 25.0% | 3 | 25.0% | 0 | 0.0% | 3 | 25.0% |

Supplementary Table 18: Association between mandatory ID consultation and the integration of OPAT programs within an antimicrobial stewardship program with OPAT settings.

| OPAT setting | Mandatory ID physician consultation before patient discharge (n=147) | | | OPAT programs fall under the responsibility of the AMS team (n=147) | | |
| --- | --- | --- | --- | --- | --- | --- |
|  | Yes | No | *P* - value | Yes | No | *P* - value |
| University teaching hospital | 71(68.3%) | 25(58.1%) | 0.211 | 56(64.4%) | 40(66.7% | 0.589 |
| Community teaching hospital | 13(12.5%) | 8(18.6%) |  | 11(12.6%) | 10(16.7%) |  |
| Nonteaching hospital | 16(15.4%) | 5(11.6%) |  | 15(17.2%) | 6(10%) |  |
| Other^£^ | 4(3.8%) | 5(11.6%) |  | 5(5.7%) | 4(6.7%) |  |

^£^community team, general hospital, teaching district general hospital, community admission avoidance rapid response, cure hospital, private independent provider, tertiary military hospital
